# Shisa6 mediates cell-type specific regulation of depression in the nucleus accumbens

**DOI:** 10.1038/s41380-021-01217-8

**Published:** 2021-07-12

**Authors:** Hee-Dae Kim, Jing Wei, Tanessa Call, Nicole Teru Quintus, Alexander J. Summers, Samantha Carotenuto, Ross Johnson, Xiaokuang Ma, Chenxi Xu, Jin G. Park, Shenfeng Qiu, Deveroux Ferguson

**Affiliations:** 1 Department of Basic Medical Sciences, University of Arizona College of Medicine-Phoenix, Phoenix, AZ USA; 2 Virginia G. Piper Biodesign Center for Personalized Diagnostics, Biodesign Institute, Arizona State University, Tempe, AZ USA

**Keywords:** Neuroscience, Depression

## Abstract

Depression is the leading cause of disability and produces enormous health and economic burdens. Current treatment approaches for depression are largely ineffective and leave more than 50% of patients symptomatic, mainly because of non-selective and broad action of antidepressants. Thus, there is an urgent need to design and develop novel therapeutics to treat depression. Given the heterogeneity and complexity of the brain, identification of molecular mechanisms within specific cell-types responsible for producing depression-like behaviors will advance development of therapies. In the reward circuitry, the nucleus accumbens (NAc) is a key brain region of depression pathophysiology, possibly based on differential activity of D1- or D2- medium spiny neurons (MSNs). Here we report a circuit- and cell-type specific molecular target for depression, *Shisa6*, recently defined as an AMPAR component, which is increased only in D1-MSNs in the NAc of susceptible mice. Using the Ribotag approach, we dissected the transcriptional profile of D1- and D2-MSNs by RNA sequencing following a mouse model of depression, chronic social defeat stress (CSDS). Bioinformatic analyses identified cell-type specific genes that may contribute to the pathogenesis of depression, including *Shisa6*. We found selective optogenetic activation of the ventral tegmental area (VTA) to NAc circuit increases *Shisa6* expression in D1-MSNs. *Shisa6* is specifically located in excitatory synapses of D1-MSNs and increases excitability of neurons, which promotes anxiety- and depression-like behaviors in mice. Cell-type and circuit-specific action of *Shisa6*, which directly modulates excitatory synapses that convey aversive information, identifies the protein as a potential rapid-antidepressant target for aberrant circuit function in depression.

## Introduction

Major depressive disorder (MDD) is a leading cause of disability with few effective treatments. More than 300 million individuals worldwide suffer from depression [1]. In the U.S., ~30 million individuals suffer from depression, which accounts for up to $60 billion dollars lost in annual productivity [2]. Current treatment approaches for depression are largely ineffective and leave more than 50% of patients symptomatic [3–5]. The development of novel antidepressant medications has been stagnant over the past several decades, with a majority of antidepressants derived from classic monoaminergic mechanisms serendipitously discovered more than half a century ago [6]. Thus, there is an urgent need to design and develop novel therapeutics to treat depression. Given the heterogeneity and complexity of the brain, identification of molecular mechanisms within specific cell-types responsible for producing depression-like behaviors will advance development of therapies for this disorder.

A significant challenge of gene expression analysis in whole brain tissue is that changes cannot be linked to a single cell-type, and gene expression changes that are opposing in different cell-types can be difficult to detect because these changes are averaged across whole populations [7]. Cell-type specific analysis using the RiboTag (RT) approach provides a higher level of resolution and selectivity required to address these limitations by providing actively translated transcriptomic information [8, 9]. The nucleus accumbens (NAc), a key brain reward region, is most often associated with rewarding and motivational effects, which are reduced in depression. More than 95% of neurons in the NAc are primarily composed of two medium spiny neuronal (MSN) subtypes enriched with dopamine D1 or D2 receptors, which have opposing roles in reward, action-value, reinforcement, and responses to drugs of abuse [10–12]. Specifically, the RT approach is advantageous for gene expression analysis of adjacent, morphologically indistinguishable D1- and D2-MSNs in the NAc. Using the RT approach, we dissected the transcriptional profile of both MSNs following chronic social defeat stress (CSDS) model of depression. Results from this study revealed that *Shisa6* contributes to the cell-type-specific molecular genetic pathogenesis of depression.

## Materials and methods

### Animals

Male C57BL/6J mice (7–9 weeks old) were obtained from Jackson Laboratory and housed on a 12 h light-dark cycle with access to food and water ad libitum. Male CD1 retired breeder mice (9–13 months old) were obtained from Charles River Laboratories. Mice acclimated to the facility for 1 week before any experimentation. D1-Cre hemizygote (line FK150) or D2-Cre hemizygote (line ER44) BAC transgenic mice from GENSAT [13] on a C57BL/6J background were used for behavioral experiments. To induce Rpl22-HA in either D1 or D2 neurons, we crossed homozygous RT mice [8] with D1 or D2 heterozygous mice. To label D1 neurons for electrophysiological recordings, Ai6 mice were crossed with D1-Cre mice (Ai6; D1-Cre). All behavioral tests and animal sacrifices (tissue collections and acute brain slice preparations) were performed at ZT 02-06. All animal procedures were approved by the University of Arizona Medical School Institutional Animal Care and Use Committees.

### Chronic social defeat stress

Social defeat stress was performed according to published protocols [14, 15]. Test mice were exposed to an unfamiliar and aggressive (<1 min latency to start aggressive behaviors) male CD1 retired breeder mouse for 5 min per day for up to 10 days. Following direct interaction with the CD1 aggressor, animals were placed in an adjacent compartment of the same cage for the next 24 h with sensory, but not physical contact. Control animals were housed in equivalent cages but with members of the same strain. 24 h following the last social defeat, animals were assayed on the social interaction (SI) test and sorted into either susceptible or unsusceptible (resilient) phenotypes based on interaction scores. Briefly, for the SI test in the first 2.5 min trial (“target absent”), the test mouse was allowed to explore freely a square-shaped open-field arena (44 × 44 cm) possessing an empty wire-mesh cage (10 × 6 cm) opposed to one side. During the second 2.5 min trial (“target present”), the mouse was reintroduced into this arena now containing an unfamiliar CD1 mouse within the smaller cage. EthoVision video-tracking software (Noldus, Leesburg, VA) was employed to measure time spent in the “interaction zone” (IZ) (14 × 26 cm). The SI ratio was calculated as 100 × (IZ time of “target present”)/(IZ time of “target absent”) [14–16]. Mice showing <100% of SI ratio are grouped as depression-susceptible mice, and mice showing over 100% SI ratio belong to depression-resilient group. For the transcriptome analysis, we identified distinct resilient (Res) and susceptible (Sus) mice from several batches of CSDS considering both IZ and corner zone time (CZ) (Res: increased IZ/decreased CZ, Sus: decreased IZ/increased CZ in the presence of “target” compared to “no target” session).

### Sub-maximal social defeat stress

To test whether an experimental manipulation might potentiate an animal’s susceptibility to defeat stress, we used a sub-maximal social defeat procedure, in which mice were subjected to repeated defeat episodes over the course of 1 day [15, 16]. Animals were placed into the CD1 aggressor’s cage for 5 min, followed by a 15 min break. This was repeated two further times with different CD1 aggressors.

### RiboTag immunoprecipitation

Six NAc tissue punches (2 mm) from each RT; D1-Cre or RT; D2-Cre mouse are homogenized in 1 ml of homogenization buffer [8] for each sample (two mice, which have similar SI ratio, pooled for each sample). Samples are then centrifuged at 4 °C, 10,000 *g* for 10 min and 800 μl of clear supernatants are mixed with 5 μg of HA-antibody (Abcam, Cambridge, MA), and incubated on a gentle rotator at 4 °C overnight. The next day, 200 μl of magnetic beads (Dynabeads anti-rabbit IgG; Life technology, Carlsbad, CA) are added and incubated overnight with constant rotation. The following day, magnetic beads are washed three times in the magnetic rack for 10 min with a high salt wash buffer. Each RNA sample was extracted using the Direct-zol miniprep kit (Zymo Research, Irvine, CA) and subjected to qRT-PCR or RNA-seq. For quality control, RNA integrity (RIN) values were measured and samples with RIN < 7 were excluded.

### Library preparation and RNA-seq

RNA-seq was performed by UAGC (University of Arizona Genetics Core, University of Arizona, Tucson, AZ). Briefly, purified RNA samples from RT immunoprecipitation (500 ng of RNA for each) were prepped for libraries after poly-A selection (KAPA Stranded mRNA-Seq Kit; Roche, Indianapolis, IN) and the libraries were sequenced in NextSeq500 (Illumina, San Diego, CA). Randomly chosen samples were divided into four sets and run in high output mode (maximum 400 million reads, 2 × 150 bp paired end reads) for each set. All the samples were normalized before pooling and loading on the sequencer.

### RNA-seq data analysis

Data were analyzed by Rosalind (https://rosalind.onramp.bio/), with a HyperScale architecture developed by OnRamp BioInformatics, Inc. (San Diego, CA). Reads were trimmed using cutadapt. Quality scores were assessed using FastQC. Reads were aligned to the Mus musculus genome (mm10) using STAR. Individual sample reads were quantified using HTseq and normalized via Relative Log Expression (RLE) using DESeq2 R library. Read distribution percentages, violin plots, identity heatmaps, and sample MDS plots were generated as part of the QC step using RSeQC. DEseq2 was also used to calculate fold changes and *p* values. Clustering of genes for the final heatmap of differentially expressed genes (DEGs) was done using the Partitioning Around Medoids (PAM) method using the fpc R library. Functional enrichment analysis of pathways, gene ontology, domain structure, and other ontologies was performed using HOMER. Several database sources were referenced for enrichment analysis, including Interpro, NCBI, KEGG, MSigDB, REACTOME, WikiPathways. Enrichment was calculated relative to a set of background genes relevant to the experiment. Additional gene enrichment analyses were performed using Enrichr [17]. Heatmaps were generated with Orange (version 3.20.1) and additional statistical analyses were performed using Prism (version 8.3.0; GraphPad Software). Meta-analysis between different datasets was performed using Galaxy. The sequencing data were imported to the Galaxy web platform, and the public server at usegalaxy.org was used to analyze the data.

### Immunohistochemistry

Mice were given a lethal dose of Euthasol (Virbac, Westlake, TX), then sequentially perfused with PBS and 4% paraformaldehyde. For eGRASP experiments, brains were fixed 8 weeks after viral injection to ensure robust expression of viral vectors at the nerve ends in the NAc. Brains were post-fixed in 4% paraformaldehyde overnight and cryoprotected in 30% sucrose solution at 4 °C. After equilibration in the sucrose solution, brains were sectioned at 30 μm on a sliding block microtome (American Optical). Free-floating sections were washed with PBS, then blocked (3% donkey serum, 0.1% Triton X-100 in PBS). The following primary antibodies were applied overnight at 4 °C with constant agitation: anti-CRE (mouse monoclonal, 1:500; Millipore, Burlington, MA), anti-HA (rabbit polyclonal, 1:2000; Abcam), anti-NeuN (mouse monoclonal, 1:500; Millipore), anti-GFAP (mouse monoclonal, 1:1000; Santa Cruz, Dallas, TX), and anti-2A peptide (mouse monoclonal, 1:200; Millipore). After 4 °C overnight incubation with constant agitation, sections were incubated with appropriate secondary antibodies conjugated with fluorescent dyes (Alexa 488, Alexa 555, and Cy5; Life technology) for 2 h at room temperature, after which they were washed, sections were mounted with Vectashield containing DAPI (Vector Labs, Burlingame, CA) and analyzed using confocal microscopy at various magnifications (LSM710; Carl Zeiss, San Diego, CA).

### In situ hybridization

Mice were given a lethal dose of Euthasol (Virbac), then sequentially perfused with PBS and 4% paraformaldehyde. Brains were post-fixed in 4% paraformaldehyde overnight and cryoprotected in 30% sucrose solution at 4 °C. After equilibration in the sucrose solution, brains were sectioned at 15 μm on a sliding block microtome (American Optical). Free-floating sections were mounted on slides and baked for 1 h at 60 °C. Probes were hybridized for 2 h at 40 °C. Amplification steps were followed by manufacturer’s instruction (RNAscope; ACD, Newark, CA). Samples were counterstained with DAPI and covered with Vectashield mounting solution (Vector Labs). Signals were imaged using confocal microscopy at various magnifications (LSM710; Carl Zeiss) and processed by Fiji (ImageJ; NIH, Bethesda, MD). For colocalization and expression analyses of *Shisa6* in D1-MSNs, all images were binary transformed and noise reduced by “Despeckle” and “Remove outlier” functions in ImageJ. Each corresponding Drd1 image was used as a region of interest (ROI; boundaries of D1-neurons) template to calculate cell-type specific expression using the “Analyze particles” function. The total Shisa6 fluorescence signal in the ROI is equivalent to the total Shisa6 expression level of Shisa6^+^ Drd1^+^ cells. Collected data from ImageJ were analyzed for two criteria: % colocalization = number of Shisa6^+^ Drd1^+^ cells/number of Drd1^+^ cells, average expression level of Shisa6^+^ Drd1^+^ cells = total expression level of ROI/number of Shisa6^+^ Drd1^+^ cells.

### Adeno-associated viruses (AAVs) and stereotaxic surgery

Cre-dependent AAV vectors (serotype 5; double inverted open reading frame (DIO) AAV construct) expressing mouse Shisa6, mCherry or shRNAs (for Shisa6 or scrambled) were cloned and packaged by VectorBuilder (Chicago, IL): AAV-DIO-Shisa6-T2A-mCherry (6.55 × 10^13^(gc/ml)), AAV-DIO-mCherry (2.33 × 10^13^), AAV-DIO-Shisa6 shRNA (4.56 × 10^13^) and AAV-DIO-scrambled (1.95 × 10^13^). The *Shisa6* knock-down target sequences are as follows: shRNA #1, 5ʹ-GCCCACTTGCCTCCATCATAT-3ʹ (1117-1137); shRNA #2, 5ʹ-AGTATAATCATCCGATCTTAA-3ʹ (989-1009); shRNA #3, 5ʹ-GGTGGAGCAGTTACACTATAT-3ʹ (1668–1688). Mice at the age of 7 weeks were anesthetized with a ketamine/xylazine mixture (ketamine 100 mg/kg and xylazine 10 mg/kg) and prepared for stereotaxic surgery. Thirty-three gauge syringe needles (Hamilton) were used to bilaterally infuse 0.5 µl of AAVs into the NAc (AP: 1.5, ML: 1.5, DV: −4.4, angle: 10°). For the eGRASP experiments, two different pre-eGRASP (cyan and yellow) and one post-eGRASP were obtained from Addgene (#111579, 111580, and 111581) and packaged in AAV vectors (serotype 5, titers: 2.06 × 10^11^, 2.01 × 10^11^, and 1.33 × 10^11^, respectively) by VectorBuilder. The cyan pre-eGRASP and yellow pre-eGRASP AAVs (0.5 µl, each site) were injected bilaterally in mPFC (AP: 1.7, ML: 0.75, DV: −2.5, angle: 15°) and vHIP (AP: −3.7, ML: 3.0, DV: −4.8, angle: 0°), respectively. The post-eGRASP AAV was injected in the NAc (AP: 1.5, ML: 1.5, DV: −4.4, angle: 10°) bilaterally (AAV-DIO-Shisa6-T2A-mCherry was injected together in case of Shisa6 colabeling eGRASP experiments). For optogenetic activation of VTA afferent inputs in the NAc, retroAAV (rAAV)-Cre (serotype rg, 7 × 10^12^) and AAV-DIO-ChR2(H134R)-EYFP (or -EYFP control; serotype 5, ChR2: 4.8 × 10^12^, EYFP: 6.5 × 10^12^; UNC Vector Core, Chapel Hill, NC) were injected in the NAc and VTA, respectively (NAc, AP: 1.5, ML: 1.5, DV: −4.4, angle: 10°; VTA, AP: −3.2, ML: 1.0, DV: −4.6, angle: 7°). Eight weeks after the viral injection, cannulae (custom made; LC 1.25 mm OD multimode ceramic zirconia ferrules (PFP), optic fiber (ThorLabs, Newton, NJ): 105 um core, 0.22 NA, Low-OH, 400–2400 nm) were implanted bilaterally in the NAc (AP: 1.5, ML: 1.5, DV: −4.0, angle: 10°) and permanently fixed to the skull with Loctite skull adhesive (Henkel, Westlake, OH). After adhesive drying, cannulas were permanently secured to the skull with Metabond (Parkell, Edgewood, NY) and dental cement (Lang Dental, Wheeling, IL). A 3% hydrogen peroxide solution was used as a wound antiseptic.

### Electrophysiological recording and morphological analysis

Whole cell patch clamp recording was conducted in D1 type medium spiny neurons (MSNs) in the NAc, followed by post-hoc neuron morphometric analysis. To overexpress Shisa6 in D1 type of MSNs, AAV-DIO-Shisa6-T2A-mCherry or control AAV-DIO-mCherry were injected into the NAc (AP: 1.5, ML: 1.5, DV: −4.4, angle: 10°) bilaterally into the Ai6; D1-Cre mice. Three weeks after injection, mice were sacrificed and the brains were quickly dissected. 350 μm coronal slices were made at the level of NAc on a vibratome (VT1200S; Leica, Allendale, NJ) in ice-cold ACSF (containing in mM, 126 NaCl, 2.5 KCl, 26 NaHCO_3_, 2 CaCl_2_, 1 MgCl_2_, 1.25 NaH_2_PO_4_, and 10 glucose saturated with 95% O_2_ and 5% CO_2_). Slices were then incubated at 32 °C for 30 min before transferred to a customized recording chamber that is mounted on a microscope (BX-51WI; Olympus, Center Valley, PA) equipped with water immersion objective (60X, NA 0.9). AAV-transduced MSNs were visualized under brightfield DIC and epifluorescence illumination. To target MSNs for whole cell recordings, neurons with soma at least 50 μm below the slice surface were selected to minimize truncation of dendritic structure. The internal electrode solution contains (in mM): 130 K-gluconate, 10 HEPES, 4 KCl, 0.3 GTP-Na, 4 ATP-Mg, 2 NaCl,1 EGTA and 14 phosphocreatine (pH 7.2, 295–300 mOsm) when used for spontaneous EPSCs recordings and for action potentials evoked by current steps. The internal electrode solution for spontaneous IPSCs recording contains (in mM): 125 KCl, 10 HEPES, 2 MgCl_2_, 0.3 GTP-Na, 2 ATP-Mg, 2.8 NaCl and 0.6 EGTA (pH 7.2, 295–300 mOsm). Spontaneous IPSCs were recorded in ACSF supplemented with CNQX (20 M; Tocris, Minneapolis, MN). To record stimulus-evoked synaptic responses and quantify the AMPA/NMDA receptor current ratio, the electrode internal solution contains (in mM): 125 Cs-gluconate, 5 TEA-Cl, 10 HEPES, 2.5 CsCl, 8 NaCl, 5 QX314-HCl, 4 Mg^2+^-ATP, 0.3 Na_3_GTP, 1 EGTA and 10 phosphocreatine. In addition, 50 μM picrotoxin was added to the perfusate ACSF to block inhibitory currents. Monosynaptic responses were obtained while MSN neurons were voltage clamped at −70 mV (AMPAR-mediated synaptic currents) or +40 mV (AMPAR + NMDAR). A bipolar stimulating electrode (FHC) was placed at ~100 mm away from the soma, and a stimulus isolator (Iso-flex, AMPI) was used to deliver a bipolar electrical stimulus.

To visualize the morphology of the recorded MSN neurons, 0.15% (W/V) biocytin was included in the electrode internal solution. Neuronal signal was amplified using a MultiClamp 700B patch clamp amplifier (Molecular Devices, Forster City, CA), low-pass filtered at 1 kHz (voltage clamp) or 10 kHz (current clamp) and digitized at 20 kHz using a Digidata 1440 A board under control of pClamp 10.6 (Molecular Devices, San Jose, CA). Only neurons in the NAc shell, which were double labeled with both ZsGreen1 and mCherry, were recorded. To test voltage responses to current injections, incremental current steps (−100 to 300 pA in 50pA increment) was used. To reconstruct the morphology of the recorded MSNs, slices were fixed in 4% PFA overnight, and incubated in avidin-Alexa 488 (Life technology) for 24 h in PBS containing 0.2% Triton X-100. Slices were then extensively washed, mounted on slides with two 350 μm-thick spacers to prevent collapsing the tissue. Dendritic arborization was visualized on a microscope (Axophot; Carl Zeiss) equipped with fluorescent light source. Neurites were traced in three-dimension using Neurolucida 360 (MicroBrightField, Williston, VT) system. After the entire arborization was traced, morphometric analysis was conducted using Neurolucida Explorer. Sholl analysis was performed to quantify the dendritic length and number of intersections at various distances from soma.

### Optogenetic stimulation

Optic fiber implanted mice expressing optogenetic tools (AAV-DIO-ChR2 or -EYFP control) are prepared in advance (see AAVs and stereotaxic surgery). Chronic optogenetic stimulation started a week after the cannulation surgery. Optic fiber patch cords (ThorLabs; optic fiber: 105 um core, 0.22 NA, Low-OH, 400–2400 nm) attached with implanted optic cannulas and rotary joint (ThorLabs; to ensure free movement of mice) are connected through an FC/PC adaptor to a 473 nm blue laser diode (Opto Engine LLC, Midvale, UT) and light pulses are generated through a waveform generator (Agilent, Santa Clara, CA). Laser power is controlled to be 3–5 mW from the tip of the fiber. Subject mice were placed in their home cages and blue light (473 nm) phasic pulses with 20 Hz for 40 ms were delivered for 5 min per day for 10 days.

### Behavior tests

All subject mice were placed in the testing room 1 h before testing for acclimation. Data were analyzed in time bins of 2.5 min (1st and 2nd half; labeled) and full time (5 min) and significant results were presented (otherwise, full time results were presented).

#### Open-field test

Open-field assessments, which are thought to reflect anxiety-related behavior, were conducted in arenas similar to those used for the SI tests (without small cage enclosures) under red light. EthoVision video tracking-based methods (Noldus) were used to record the distance traveled and the time spent in the open arena and a delineated “center zone”.

#### Elevated-plus maze (EPM)

The elevated-plus maze, an assay of anxiety-like behavior, consisted of two opposite open arms (60 × 15 cm) and two enclosed arms (60 × 15 cm, surrounded by a 15 cm-high black wall) elevated 75 cm from the ground. Animals were placed at the center of the maze for each individual trial lasting 5 min under red light. Number of entries and time spent in open arms was measured in addition to the number of entries in enclosed arms.

#### Light-dark box test (LDT)

The light-dark box tests, an assay of anxiety-like behavior, were conducted in the same arenas used for the open-field test. A closed black box (44 × 15 × 30 cm) which has a small open door (7 × 7 cm), was placed in one side of the arena. Animals were placed in the light compartment and the time spending in the light compartment were analyzed with EthoVision for a period of 5 min. The experiment was performed under white light conditions.

#### Forced swim test (FST)

The forced swim test, which measures despair-like behaviors, was performed as described previously [16]. Animals were placed in a 4 L beaker containing 3 L of water at a temperature of 25 ± 1 °C. Tracking was performed with EthoVision for a period of 5 min. The experiment was performed under red light. Animals were analyzed for the amount of time-spent mobile vs immobile.

#### Tail suspension test (TST)

For the tail suspension test, which measures despair-like behaviors, each mouse was suspended with a long adhesive tape (taped 2 cm from tip of tail, ~20 cm above from a shelf bottom). Its tail was passed through a small paper cylinder (width 1.5 cm, height 2 cm) before suspension to prevent climbing. Tracking of recorded video was performed with EthoVision for a period of 5 min. The experiment was performed under white light conditions. Animals were analyzed for the amount of time-spent mobile vs immobile.

#### Sucrose preference

To assess natural reward using the sucrose preference test, 50 ml tubes containing stoppers fitted with ballpoint sipper tubes were filled with solutions containing either 1% sucrose diluted in drinking water, or drinking water alone. The weights of solutions were recorded, and the position of the tubes was interchanged daily to eliminate side bias. Sucrose preference was calculated as a percentage of sucrose intake over total fluid volume consumed and averaged over 3 days of testing.

### Statistical analysis

Statistical analyses of transcriptome data are described in the *RNA-seq data analysis* section. For all other analyses, unpaired or paired Student *t* tests, one-way or two-way ANOVAs with Tukey’s or Sidak’s post-hoc test were performed to determine significance for conditions in which there were more than two groups or two factors. Statistical analysis was performed using Prism (version 8.3.0; GraphPad, San Diego, CA). To assess the homoscedasticity and normality of the distribution, the Shapiro–Wilk normality test was performed before parametric statistical analyses. We performed power analyses based on previous data using Statmate (Graphpad). A sample size of 7 in each group of social defeat experiments has a 95% power to detect a difference between means of 42.03 (SI ratio) with a significance level (alpha) of 0.05 (two-tailed). All values included in the figure legends represent means ± s.e.m.

## Results

### D1- and D2-MSNs express distinct transcriptomes

**Fig. 1:**
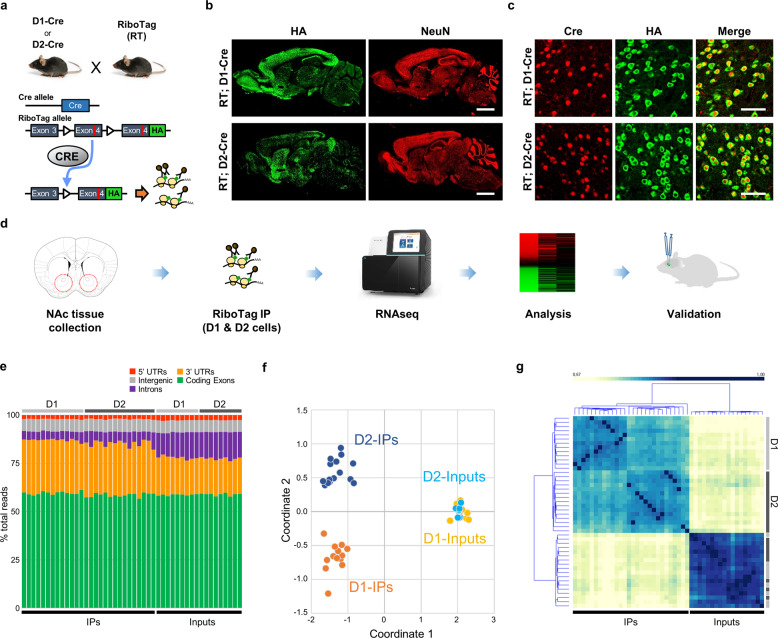
MSN subtype specific transcriptomics in the NAc. **a** Schematic diagram of RT; D1-Cre and RT; D2-Cre mice generation and purification of MSN-subtype specific transcripts. The exogenous exon 4-HA cassette is expressed when the floxed exon 4 is excised in Cre-expressing cells. The HA-tagged polyribosome allows cell-type specific mRNA preparation from D1-MSNs (RT; D1-Cre) or D2-MSNs (RT; D2-Cre). **b**, **c** Validation of RiboTag mice with immunohistochemistry across brain regions (sagittal; scale bar, 2 mm) (**b**) and in the NAc (coronal; scale bar, 50 μm) sections (**c**). **d** Schematic diagram of experimental design. **e** Read mapping composition of each RNA-seq sample on the mouse genome, mm10 (% average for coding regions: IPs, 59.2% vs inputs, 58.8%; for intronic regions: IPs, 5.5% vs inputs, 13.4%). **f** Clustering of RNA-seq samples of RT; D1- and D2-Cre in a MDS plot. **g** Sample correlation heatmap were plotted with clustering (The dendrogram annotations on the right side axis: D1, light gray; D2, dark gray). For experiments in (**e**–**g**) D1-IPs, *n* = 13; D2-IPs, *n* = 15; D1-inputs, *n* = 9; D2-inputs, *n* = 9.

D1- and D2-MSNs in the NAc have distinct and opposite roles in despair-like behavior [10–12], which suggest differential regulation of gene expression in each subtype. To evaluate the transcriptomic profile of MSNs, we generated mouse lines targeting D1- or D2-MSNs by crossing RT mice [8] with D1- or D2-Cre transgenic lines (RT; D1-Cre and RT; D2-Cre). This allows cell-type specific isolation of transcripts from D1- or D2-MSNs in the NAc (Fig. 1a). HA-tagged ribosomes express in a Cre-dependent manner and thus only a subset of neurons, expressing D1- or D2-receptors are positive for HA (Fig. 1b, c, Supplementary Fig. 1). To analyze D1 and D2 transcriptomes in an unbiased manner, RNA sequencing (RNA-seq) experiments were performed as depicted in Fig. 1d. On average, 92% of 19.3 M reads from each sample was aligned with a high Q30 value (89% bases above Q30), validating high quality of sequencing from RT samples (Supplementary Table. 1) in accordance with relatively uniform distributions of unnormalized counts (Supplementary Fig. 2a). IP samples are enriched with mature mRNA which has less intronic regions, whereas transcripts from input samples have more immature, pre-mRNAs containing introns (Fig. 1e), as reported previously [9, 18]. Normalized gene expression profiles were obtained and plotted for each sample in a multidimensional scaling (MDS) plot, which showed distinct clustering of transcriptional profiles for each MSN subtype in IP samples (Euclidean distance: 1.258), but not in inputs which contain mixed population of cell-types in the NAc (Euclidean distance: 0.062) (Fig. 1f). In addition, unsupervised sample correlation mapping shows separate clustering of D1 and D2 groups in IP samples but not in input samples (Fig. 1g). Indeed, pairwise comparisons between independent biological replicates in D1 or D2 groups show high correlation values: avg. correlation in D1, *R* = 0.994; in D2, *R* = 0.993 (Supplementary Fig. 2b, c). The results from the MDS and correlation mapping are indicative of successful isolation and sequencing of distinct MSN subtypes in the NAc.

**Fig. 2:**
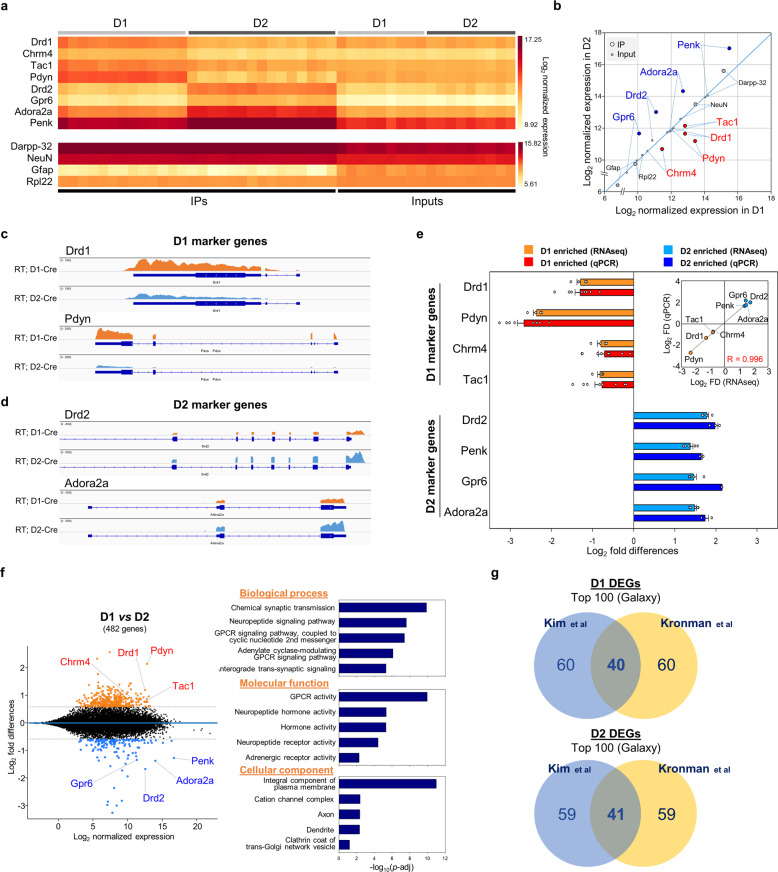
Validation of MSN subtype specific transcriptomics with D1- or D2-MSN specific marker genes. **a**, **b** Heatmap (**a**) and scatter plot (**b**) visualization of normalized expressions for MSN-subtype specific marker genes (D1-IPs, *n* = 13; D2-IPs, *n* = 15; D1-inputs, *n* = 9; D2-inputs, *n* = 9). **c**, **d** Representative aligned reads on selected D1- (*Drd1* and *Pdyn*) (**c**) or D2-marker genes (*Drd2* and *Adora2a*) (**d**) were visualized and compared by IGV. **e** Fold differences of D1- or D2-marker gene expressions from RNA-seq (D1 enriched, *n* = 4; D2 enriched, *n* = 5) were plotted with independent qPCR validation data (D1 enriched, *n* = 8; D2 enriched, *n* = 3) in bar chart (**e**; mean ± s.e.m.) and scatter plot (inset; Pearson’s correlation, *R* = 0.9961). **f** MA plot of 482 DEGs between D1 control and D2 control comparison (D1 enriched (orange dots), 339 genes; D2 enriched (blue dots), 143 genes; [Fold change (FC)] > 1.5, adjusted *p* value < 0.05). Gene ontology (GO) analysis of the 482 DEGs from the control group comparison. **g** Comparisons between top 100 D1- or D2-enriched gene lists from Kim et al. and Kronman et al. by the same RNA-seq analysis workflow in Galaxy.

Next, to validate that NAc MSNs were isolated into distinct neuronal subtypes, we analyzed expression of well-characterized D1- and D2-MSN gene markers [19–22] and found enrichment of marker genes in each subtype as depicted in the heatmap and scatter plot (IP samples of RT; D1-Cre: *Drd1* (2.4×), *Chrm4* (1.8×), *Tac1* (1.7×), and *Pdyn* (5.1×); RT;D2-Cre: *Drd2* (3.6×), *Gpr6* (2.8×), *Adora2a* (2.8×), and *Penk* (2.7×)) (Fig. 2a, b). MSN (*Darpp-32*) and neuron (*NeuN*) specific genes are enriched in both D1 and D2 samples at similar levels, whereas a glia-specific marker, *Gfap*, is depleted from IP samples by 90% relative to inputs, demonstrating enrichment of neuron specific genes. Visualized read coverages for selected marker genes in D1 and D2 samples by Integrated Genomics Viewer (IGV) [23] clearly illustrates cell-type specific gene enrichment (Fig. 2c, d), whereas nonneuronal markers are depleted in the IP samples (Supplementary Fig. 3). These results were further validated by quantitative PCR (qPCR) using independent biological samples (Fig. 2e), which validate the reproducibility of RNA-seq quantification using RT and demonstrate our ability to efficiently segregate D1- and D2-MSNs with minimal cross-contamination.

We determined that there were 482 DEGs between D1- and D2-MSNs ([Fold change (FC)] > 1.5, adjusted *p* value < 0.05), GO analysis of the 482 DEGs revealed that gene modules involving G-protein coupled receptors, synaptic transmission and neuropeptide related gene sets were enriched (Fig. 2f, Supplementary Table. 2). We analyzed the reproducibility of our results by comparing our dataset with a recently published report using the same RT method in the NAc [24]. To minimize inter-data variations between our results (Kim et al.) and Kronman’s [24], we analyzed and compared both datasets using the same analysis workflow in Galaxy [25]. We found approximately a 40% overlap in gene expression in both D1 and D2 (top 100) genes between Kronman et al. [24] and our dataset (Fig. 2g), which demonstrates concordance between the two studies.

As the opposing roles of D1- and D2-MSNs are important to understand the neurobiology of depression [10–12], we further analyzed D1 and D2 transcriptomes in order to detect novel MSN subtype specific genes. Toward this end, we compared our data with two different MSN transcriptome datasets; one from RNA-seq analysis using RT [24] and the other a BAC-TRAP based microarray experiment [21] (Supplementary Fig. 4a). Overall, nearly a quarter of genes (D1, 93/339: 27.4%; D2, 36/143: 25.2%) from our datasets are shared with both studies, including all D1 and D2 marker genes tested in Fig. 2. More importantly, there are other, uniquely detected genes from both D1- (246 genes) and D2-comparisons (107 genes) in our datasets (Supplementary Fig. 4a). Selected genes from the lists, sorted by fold differences and adjusted *p* values, show strong correlation between RNA-seq and qPCR (Pearson’s correlation, *R* = 0.966), which were further validated with in situ hybridization for *Oxtr*, *Kcnj3*, *Ntrk1*, and *Tacr1* (Supplementary Fig. 4b–e). These results establish the validity of RT to detect novel cell-type specific mRNA transcripts and provide information on expression patterns at single cell levels, implicating transcriptional variability within subpopulations of each MSN subtype (Supplementary Fig. 4e).

### D1 transcriptome correlates with susceptibility

**Fig. 3:**
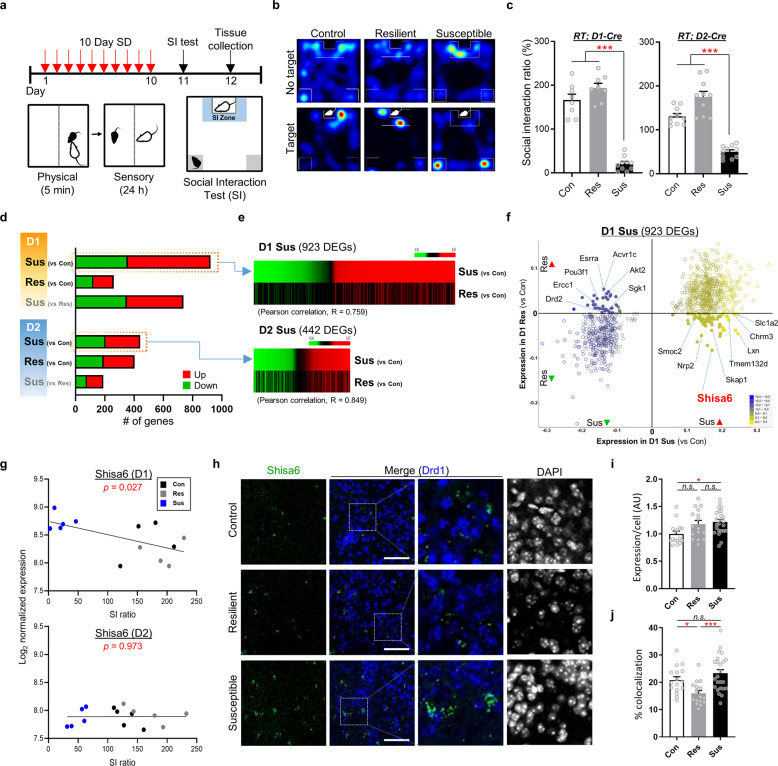
Stress group-specific changes of gene expression in D1- and D2-MSNs. **a**, **b** Schematics of chronic social defeat stress (CSDS) (**a**) and representative heat maps of social interaction (SI) behavior (**b**). **c** SI ratio of RiboTag (RT); D1- or D2-Cre mice which were used for RNA-seq analysis (vs control or resilient, ***p < 0.001; RT;D1-Cre, *F*_(2,23)_ = 100.9; RT;D2-Cre, *F*_(2,27)_ = 54.63; calculated by one-way analysis of variance (ANOVA) with Tukey’s post-hoc test; D1: Con, *n* = 8; Res, *n* = 8; Sus, *n* = 10; D2: Con, *n* = 10; Res, *n* = 10; Sus, *n* = 10). **d** Numbers of DEGs from comparisons between stress groups are plotted (threshold, *p* < 0.05; # of DEGs for D1 comparisons: Sus vs Con, 923 (566 up; 357 down), Res vs Con, 261 (138 up; 123 down) and Sus vs Res, 737 (386 up; 351 down); for D2, Sus vs Con, 442 (237 up; 205 down); Res vs Con, 403 (211 up; 192 down) and Sus vs Res, 190 (111 up; 79 down)). **e** D1- and D2-susceptible DEGs (vs control, *p* < 0.05) are sorted by fold change (FC) values and compared to FC values of corresponding resilient DEGs (vs control, *p* < 0.05). **f** A scatter plot of D1-susceptible DEGs with log2 fold difference values shows inversely modulated genes (filled circles, 102 genes) in susceptible and resilient groups. **g** *Shisa6*, one of the 102 genes, inversely correlates with SI ratio in D1, but not D2. *p* values in linear regression. **h**–**j** Validation of *Shisa6* expression in the NAc of socially defeated mice using in situ hybridization (RNAscope; scale bar, 100 μm) (**h**). Average expression at single cell level (**i**) and % colocalization (**j**) of Shisa6^+^ cells in D1-MSNs (Drd1^+^) (mean ± s.e.m.; *p < 0.05, ***p < 0.001; n.s. not significant; expression/cell, *F*_(2,53)_ = 4.705; % colocalization, *F*_(2,53)_ = 8.507; calculated by one-way ANOVA with Tukey’s post-hoc test). For experiments in (**d**, **g**) D1 Con, *n* = 4; D1 Res, *n* = 4; D1 Sus, *n* = 5; D2 Con, *n* = 5; D2 Res, *n* = 5; D2 Sus, *n* = 5. For experiments in (**i**, **j**) Con, *n* = 16; Res, *n* = 16; Sus, *n* = 24 from 4 Con, 4 Res, and 6 Sus mice.

To determine the transcriptional networks differentially regulated by stress in D1- and D2-MSNs, we applied CSDS to RT lines (RT; D1 Cre and RT; D2 Cre) for 10 days and their susceptibility or resilience to chronic stress was assessed using the SI test [14, 15] (Fig. 3a, b). Susceptible groups in both RT lines exhibit significant decreases of SI ratio (Fig. 3c; RT; D1-Cre, *F*_(2,23)_ = 100.9, *p* < 0.001 (4.09E−12); RT; D2-Cre, *F*_(2,27)_ = 54.63, *p* < 0.001 (3.23E−10)), while their locomotor activity levels were not changed (Supplementary Fig. 5). For transcriptomic analysis, we combined tissue from two mice which show similar levels of SI ratio, and performed RT immunoprecipitation followed by RNA-seq. D1 and D2 transcriptomes from each group (control, Res and Sus) were analyzed to detect DEGs for Sus vs Con, Res vs Con, or Sus vs Res with DeSeq2 (*p* value < 0.05, vs control or Res) (Fig. 3d). Interestingly, within the D1 dataset, Sus group (vs Con, 923 DEGs) show approximately three times more DEGs than Res group (vs Con, 261 DEGs), whereas in the D2 dataset both Sus (vs Con, 442 DEGs) and Res group (vs Con, 403 DEGs) show similar number of DEGs (Fig. 3d, Supplementary Tables 3–8). Indeed, the third DEG set, Sus vs Res, in D1 shows 737 genes were differentially regulated, but the number is only 190 in D2, which indicates that Sus and Res transcriptome profiles are very different in D1, and relatively similar in D2. In addition, we compared the three different DEG lists for each cell-type in venn diagrams and found more genes are shared between Sus (vs Con) and Sus (vs Res) in D1, whereas, in D2, Sus (vs Con) and Res (vs Con) show more overlapped genes than Sus (vs Con) and Sus (vs Res) (Supplementary Fig. 6a, c). We hypothesized that genes showing distinct transcriptional profiles between Sus and Res are important in defining the Sus phenotype. Therefore, we created parallel plot heatmaps for Sus (vs Con) DEGs and compared Sus and Res profiles in each cell-type (Fig. 3e, Supplementary Fig. 6b, d). Broad patterns of transcriptional profiles show similar patterns in the D2 plot, and rather distinct in the D1 (Fig. 3e). For the D1 Sus DEGs (923 genes), only 73 genes (7.9%) exhibited significant changes in the same direction (*p* < 0.05) in the D1 Res, whereas in the D2 Sus DEGs (442 genes), more genes (85 genes, 19.2%) were changed also in D2 Res (*p* < 0.05). Taken together, in terms of gene numbers and contents of the DEG lists, the D1 transcriptome, but not D2 transcriptome, shows distinct DEG profiles for Sus and Res (Pearson’s correlation in D1, *R* = 0.759; in D2, *R* = 0.849) (Fig. 3e).

We hypothesized that genes showing opposite expression patterns between Sus and Res in D1 may play a role in the Sus phenotype. To identify the genes, we created a scatter plot from log2 fold difference data of D1 Sus (vs Con) and D1 Res (vs Con) (Fig. 3f). We found a total of 102 genes regulated in opposite directions in Sus and Res groups in the D1 dataset, whereas only 13 genes, mostly with marginal changes, were found in the D2 (Fig. 3f, Supplementary Fig. 6e, f). To determine their potential contribution to behavioral pathology, we performed GO analysis for D1 genes (102 genes) which revealed terms associated with neuronal function including synapse (*p* = 3.18E−08), dopaminergic- (*p* = 7.07E−04) and GABAergic-synaptic transmissions (*p* = 1.37E−03) (Supplementary Fig. 6h). Moreover, when compared to a curated gene set for depressive disorder (Harmonizome [26]), ~40% of genes overlapped with the gene set for depressive disorders (Supplementary Fig. 6g), suggesting that the 102 genes contain potential target genes functionally associated with susceptibility.

From the D1 Sus gene list, we focused on *Shisa6* which showed increased expression in Sus, and decreased in Res, these changes were the largest among the 102 genes (Fig. 3f). Furthermore, from D1 vs D2 comparisons (Supplementary Fig. 7, Supplementary Table. 2, 9, 10), we also found *Shisa6* in the D1 Sus DEG list, which is associated with highly relevant GO terms such as MDD, bipolar disorder, and anxiety in a GWAS database (Supplementary Fig. 7d). These data suggest that *Shisa6* may function as a potential pro-depressive gene in D1-MSNs. *Shisa6* was recently identified as a component of AMPA receptors (AMPAR), which is expressed in various brain regions (Supplementary Fig. 8), and sustains AMPAR currents by preventing desensitization [27]. Recently, *Shisa6* was found in a proteome analysis of developing synapses [28] in addition to facilitating purkinje cell synaptic excitability and procedural memory [29]. Interestingly, *Shisa9*, which has opposite functions to *Shisa6* at glutamatergic synapses [27], was identified as a highly associated gene for major depression in recently published large-scale GWAS studies [30, 31]. Here we show a strong inverse correlation between *Shisa6* expression and SI solely in D1-MSNs (*p* = 0.027), but not in the D2-MSNs (*p* = 0.973) (Fig. 3g). We further validated the inverse correlation between *Shisa6* expression in D1 and SI with in situ hybridization assay using an independent social defeat cohort, which showed higher expression of *Shisa6* in D1-MSNs of Sus mice (Fig. 3h–j). Only the Sus group showed a significant increase of *Shisa6* expression by 22% in each cell (Fig. 3i), which corresponds to RNA-seq data (Fig. 3g). Shisa6^+^ D1 cells in Sus mice (23.3%) show similar % colocalization with the Con (20.8%), and only the Res group (15.9%) exhibits significant decrease in colocalization (Fig. 3j), which implicates only small subsets of D1-MSNs express *Shisa6*, and the *Shisa6* expression is controlled in the restricted D1 populations according to Sus or Res phenotype.

### Shisa6 functions in a circuit-specific manner

**Fig. 4:**
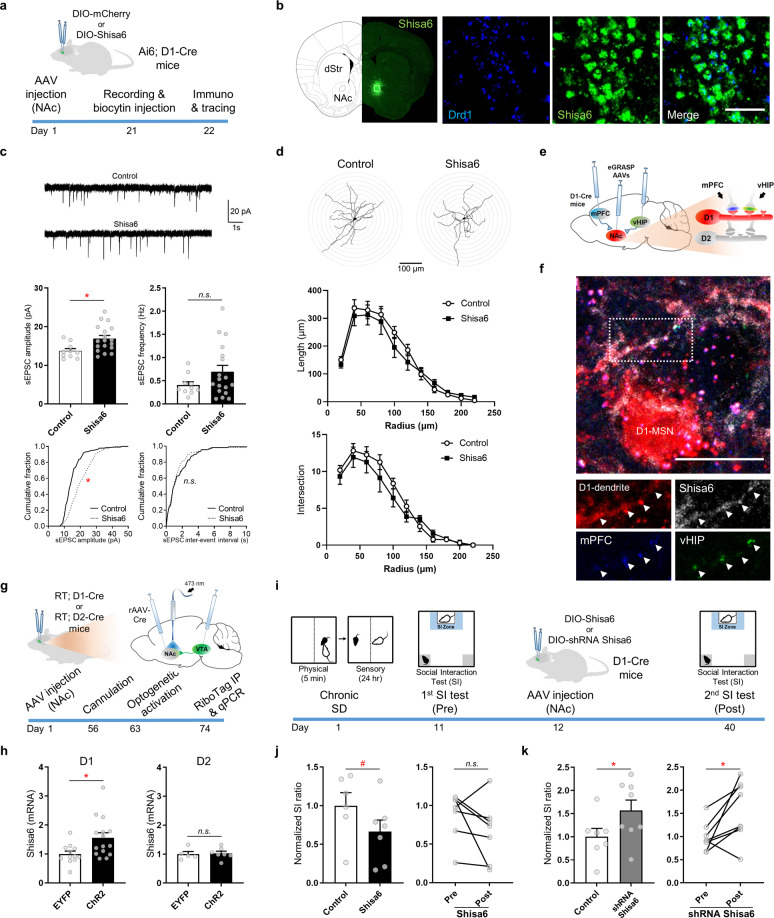
Functional validation of Shisa6 related to depression phenotypes. **a** Schematics of experimental plan of electrophysiological and morphological analyses. **b** Validation of D1-MSN specific viral expression of *Shisa6* (in situ hybridization; scale bar, 50 μm). **c** Representative sEPSC traces, bar graphs (mean ± s.e.m.) and cumulative distribution of sEPSC amplitude and frequency in D1-MSN from control vs Shisa6 comparisons (amplitude: control, 14.4 ± 0.8 pA; Shisa6, 16.9 ± 0.8 pA; frequency: control, 0.41 ± 0.07 Hz; Shisa6: 0.69 ± 0.13 Hz; control, *n* = 10; Shisa6, *n* = 18). **d** Morphological analysis of biocytin-injected control or Shisa6-overexpressing D1-MSNs (control, *n* = 11; Shisa6, *n* = 15). **e**, **f** Afferent input specific excitatory synapse labeling (eGRASP) and *Shisa6* colabeling in D1-MSNs. Schematics of D1-MSN specific eGRASP in the NAc (**e**) and colocalization of Shisa6 at mPFC and vHIP afferent synapses (**f**; arrowhead: mPFC or vHIP eGRASP, scale bar: 20 μm). **g**, **h** Chronic optogenetic activation of VTA to NAc circuits and MSN subtype specific Shisa6 gene expression analysis. **g** Schematics of optogenetic experiments. VTA to NAc circuit of RT; D1-Cre or RT; D2-Cre mice was labeled with retrograde Cre-AAV (rAAV2-Cre in the NAc) and Cre-dependent ChR2 or EYFP AAVs (in the VTA) and then implanted with optic-cannula in the NAc. Mice received optogenetic activation (20 Hz, phasic 473 nm, for 5 min/day) for 10 days. **h** RiboTag IP and qPCR analysis of Shisa6 gene expression in D1- or D2-MSNs after 10 day optogenetic activation (D1: EYFP, *n* = 12; ChR2, *n* = 15; D2: EYFP, *n* = 5; ChR2, *n* = 7). **i**–**k** Behavioral analysis of D1-MSN specific Shisa6 modulation in socially defeated mice. **i** Schematics of behavioral experiments and timeline. **j**, **k** Assessments of social interaction changes after Shisa6 overexpression (**j**; control (DIO-mCherry), *n* = 6; Shisa6, *n* = 7) or knock-down (**k**; control (shRNA scrambled), *n* = 7; shRNA Shisa6, *n* = 8) in D1-MSNs. Values were normalized to the control. Graphs are represented as mean ± s.e.m. (*p < 0.05; ^#^*p* < 0.09; *n.s*. not significant; *t*-test, two-tailed (**c**, **h**), one-tailed (**j**, **k**); two-way ANOVA with Sidak’s post-hoc test (**d**)).

We validated that *Shisa6* shows increased expression in D1-MSNs of Sus mice via RNA-seq and in situ hybridization (Fig. 3g–j), which may suggest a pro-Sus role for *Shisa6* in D1-MSNs. To investigate potential causal relations of *Shisa6* expression and its pro-Sus effects, we designed a cell-type specific AAV vector to ectopically increase *Shisa6* expression only in D1-MSNs and measure subsequent changes in neural activity and behavior (Fig. 4). The AAV-DIO-*Shisa6*-mCherry (Supplementary Fig. 9) or –mCherry only control was injected in the NAc of D1-Cre mice bilaterally and D1-MSN specific expression was assessed with in situ hybridization (Fig. 4a, b). Localization of ectopically expressed *Shisa6* protein was also tested with immunohistochemistry, which shows intense localization at the membrane and neural processes of infected D1-MSNs (Supplementary Fig. 9b).

For the electrophysiological analysis, acute brain slices were prepared from *Shisa6* or control AAV injected Ai6; D1-Cre mice and the AAV infected D1-MSNs (mCherry and ZsGreen1 double-positive) were recorded (Fig. 4c, Supplementary Fig. 10). The amplitude, but not frequency, of spontaneous excitatory postsynaptic currents (sEPSCs) was significantly increased in *Shisa6*-overexpressing D1-MSNs compared with controls (Fig. 4c). In addition, AMPA/NMDA ratio was increased in the *Shisa6* overexpressing D1-MSNs (Supplementary Fig. 10b). In contrast, no significant alteration was found in the spontaneous inhibitory postsynaptic currents or action potentials evoked by current steps between control and *Shisa6* overexpressing D1 neurons (Supplementary Fig. 10c, d). We next conducted morphological analysis on the recorded D1-MSNs using biocytin injection. No significant change in dendritic complexity (length and number of intersections) was observed between control and Shisa6 groups (Fig. 4d). Taken together, these results suggest that *Shisa6* overexpression in D1-MSN induced augmented synaptic responses to glutamatergic inputs without significant alteration of dendritic structure.

Considering the role of *Shisa6* in AMPA receptor function, the increased amplitude of sEPSC (Fig. 4c) is highly indicative of a functional coupling of *Shisa6* and AMPAR currents at excitatory synapses. To examine localization of *Shisa6* in functionally relevant glutamatergic synapses in D1-MSNs, we labeled synapses receiving inputs from two major excitatory afferent sources to the NAc, the medial prefrontal cortex (mPFC) and the ventral hippocampus (vHIP), with dual-eGRASP [32] (Fig. 4e). Two different AAVs, which express post-eGRASP or *Shisa6* in a Cre-dependent manner, were injected together in the NAc of D1-Cre mice. We also injected two different pre-eGRASP AAVs, which express cyan- or yellow-eGRASP, in the mPFC and vHIP, respectively. We found sparsely or densely labeled eGRASP positive synapses throughout the NAc region (Supplementary Fig. 11). Ectopically expressed *Shisa6* is well colocalized with both excitatory synapses, receiving inputs from mPFC (cyan) and vHIP (yellow) (Fig. 4f), and these results strongly indicate a functional role for *Shisa6* in glutamatergic synapses, which may be relevant to the Sus phenotype (Fig. 4c).

It is of interest to ascertain whether upstream neural inputs to D1-MSNs in the NAc mediate *Shisa6* expression depending on Sus or Res status. Firing patterns of ventral tegmental area (VTA) dopamine neurons are important to determine susceptibility or resilience to social stress [16, 33, 34], therefore we evaluated whether activation of the VTA to NAc circuit can mediate *Shisa6* expression in D1-MSNs. Phasic activation of VTA to NAc circuit is a model known to exert pro-Sus effects in mice [33, 34], and is useful to assess pro-Sus genes in the NAc [35, 36]. To activate the VTA to NAc pathway, we specifically labeled the circuit with ChR2 (or EYFP as control) and stimulated the fiber ends in the NAc to ensure region specific activation (Fig. 4g, Supplementary Fig. 12). After 10 day-chronic activation of the circuit, we resolved cell-type specific expression of *Shisa6* with RT immunoprecipitation for both D1- and D2-MSNs. Interestingly, *Shisa6* showed approximately a 50% increase in D1-MSNs, whereas there was no change of *Shisa6* expression in D2-MSNs (Fig. 4h). These results suggest VTA neurons, which project to D1 are direct upstream modulators of *Shisa6* expression.

### Shisa6 modulates behavioral susceptibility

To investigate whether *Shisa6* can directly modify depression- and anxiety-like behaviors in vivo, we overexpressed or knocked-down the *Shisa6* gene in D1-MSNs and performed several behavioral assays. First, we examined baseline behavioral changes upon *Shisa6* overexpression in D1-MSNs of male and female mice (Supplementary Fig. 13). Baseline behavioral assessments showed increased anxiety-like behaviors (decreased center time in open-field test; *, *p* = 0.011) and anhedonia (decreased sucrose preference; *, *p* = 0.040) when *Shisa6* is overexpressed in D1-MSNs of male mice (Supplementary Fig. 13a–i). In the same baseline condition, female mice showed a similar trend of anhedonia (decreased sucrose preference; ^#^, *p* = 0.071) after *Shisa6* overexpression in D1-MSNs, while other behaviors showed decreasing trends of anxiety (increased open arm time in elevated-plus maze; ^#^, *p* = 0.054) and despair-like behaviors (decreased immobile time; ^#^, *p* = 0.073) (Supplementary Fig. 13j–q). With the male mice which overexpress *Shisa6* in D1-MSNs, we conducted an additional acute social defeat experiment to assess the effects of *Shisa6* increase prior to social stress exposure (Supplementary Fig. 13a). Interestingly, after acute social defeat stress (sub-maximal social defeat), the *Shisa6* overexpressed male mice exhibited no significant decrease of SI (Supplementary Fig. 13i). To determine if *Shisa6* acts in a pro-depressive manner in mice previously exposed to social stress, we selectively increased *Shisa6* in D1-MSNs after CSDS (Fig. 4i). Overexpression of *Shisa6* resulted in a decreased trend for SI (Fig. 4j; left panel, decreased by 35.6% (vs DIO-mCherry control), ^#^, *p* = 0.082; right panel, *Shisa6* overexpression (Pre vs Post), n.s., *p* = 0.101). Next, to assess if blocking *Shisa6* could rescue deficits in SI post CSDS, we selectively knock-down *Shisa6* by overexpressing shRNAs targeting *Shisa6* mRNA in D1-MSNs (Supplementary Fig. 9d–f). We found that SI was significantly increased in the *Shisa6* knock-down group (Fig. 4k; left panel, increased by 56.8% (vs shRNA scrambled control), *, *p* = 0.038; right panel, *Shisa6* knock-down (Pre vs Post), *p* = 0.015). These results demonstrate that *Shisa6* knock-down in D1-MSNs can directly rescue depression-like behaviors, by increasing SI.

## Discussion

Here we report the first cell-type specific transcriptomic analysis of socially defeated mice and identify novel DEGs in D1- and D2-MSNs, that may contribute to the pathophysiology of depression. We revealed that distinct subsets of genes, directly related to neuronal function, were differentially expressed in D1- and D2-MSNs of Sus mice. We identified more DEGs in the Sus group than Res in D1-MSNs, whereas similar numbers of DEGs were detected in both Sus and Res D2-MSNs. Although, cell-type specific genes linked to depression have been identified in chronic stress models [10, 37–41], transcriptome-wide stress-induced changes in specific neurons in the NAc have yet to be reported.

Gene expression patterns in Sus and Res are distinct in D1, as compared to D2, which suggest D1-MSNs mediate Sus phenotypes such as social avoidance and anhedonia, while D2-MSNs act on common stress pathways in both Sus and Res mice. We further analyzed the Sus and Res genes by comparing the same stress groups between D1- and D2-MSNs and found approximately half of the genes on the D1 lists show significant correlations to SI which highlights the importance of D1-MSNs in the regulation of the Sus phenotype (Supplementary Fig. 7). We found a group of Sus specific genes in D1-MSNs showing opposite direction of regulation when compared to Res mice (102 genes), and amongst the genes, we identified *Shisa6* as a potential candidate for the regulation of D1-MSNs in mice exposed to social stress. *Shisa6* expression levels are significantly increased in a subset of D1-MSNs and their levels are inversely correlated with the SI ratio. Interestingly, although cells in Res express *Shisa6* comparably to Sus, they are not highly colocalized with D1-MSNs, which suggest *Shisa6* in D2-MSNs or non-MSN populations act differently in Res mice. Recent single-cell RNA-seq analyses reveal subpopulations for each MSN subtypes [22, 42–44] and future studies should examine regionally defined subpopulation of MSNs (e.g., NAc shell vs core regions) [22, 45] or conduct circuit specific single cell analysis [46].

Shisa family members are single transmembrane adaptor proteins that regulate signaling pathways [47, 48] including neuronal transmission by altering glutamatergic or GABAergic-synaptic transmission through direct interactions with AMPAR [27] (*Shisa6* and *Shisa9*) or GABA_A_R [49] (*Shisa7*). Increased expression of *Shisa6* is expected to preserve synaptic AMPAR and prevent desensitization in D1-MSNs [27], which increases overall AMPAR current. Indeed, D1-MSNs in which *Shisa6* is overexpressed show increased sEPSC amplitudes without changing dendritic arborization. Moreover, the increased EPSC amplitudes are exclusively driven by AMPAR-EPSC which validate *Shisa6* mediated facilitation of AMPAR function. This is consistent with the observation that Shisa6 protein is localized to glutamatergic synapses, from two major afferent sources, mPFC and vHIP. In our in situ hybridization experiment, 20% of D1-MSNs express detectable amounts of *Shisa6* mRNAs, and it is of interest whether the *Shisa6*^+^ D1-MSN subpopulation has specific neural connections with other brain regions relevant to depressive-like phenotypes including VTA, mPFC, and vHIP. Interestingly, *Shisa9* was recently identified as a gene related to major depression by large-scale GWAS [30, 31] and is known to counteract *Shisa6* [27]. We observed that *Shisa9* showed a decreased trend of expression in D1-MSNs of Sus mice (Supplementary Fig. 8). Despite the fact that *Shisa6* expression in D1-MSNs was correlated with SI after CSDS, *Shisa6* in other cell-types might contribute to circuit level modulation of depressive-like phenotypes in the NAc.

*Shisa6* expression is regulated in a cell-type specific manner following CSDS and optogenetic experiments suggest that hyperactivity of VTA dopamine neurons projecting to the NAc is a putative upstream signal mediating *Shisa6* expression in D1-MSNs but not D2-MSNs of Sus mice. Interestingly, optogenetic activation of the VTA- NAc circuit is sufficient to alter *Shisa6* expression, but doesn’t alter SI behaviors. This result suggests that non-context dependent activation of dopamine neurons is insufficient to recruit glutamatergic inputs from the mPFC and/or vHIP to induce depressive-like phenotypes, which is supported by previous research [34, 35]. However, in stress conditions, *Shisa6* may be localized to specific synapses which receive glutamatergic inputs relevant to depressive phenotypes. In this regard, it is noteworthy that vHIP-NAc glutamatergic circuit delivers aversive information only to D1-MSN, but not D2, and increased synaptic strength of excitatory synapses directly increased depression-like behaviors, concurrent with increased AMPAR transmission [50]. These results are well correlated with our data showing D1-MSN specific increase of *Shisa6* and corresponding enhancement of excitability and depressive-like behaviors following CSDS. In the future, exploring the *Shisa6*^+^ D1-MSN population and their connectivity may provide valuable insight on mechanisms of depression.

Our data showing an increase in *Shisa6* in Sus mice is consistent with the hypothesis that increased excitability of the NAc contributes to stress vulnerability. For example, Vialou et al. showed that, in Sus mice and humans that experienced MDD, there was increased AMPA receptor function (i.e., higher GluR1:GluR2 ratio) in the NAc [51]. Similarly, the decrease in *Shisa6* in Res animals suggests that decreased glutamatergic function may be protective against impairing effects of chronic stress, which is consistent with studies demonstrating that decreased AMPA activity enhances reward-related behaviors [52, 53]. However, in contrast to our finding, it should be noted that other studies with different stress models [54–56] show a decrease in D1-MSN AMPA mediated excitation after stress. These conflicting results highlight the complex nature of NAc circuit regulation in depression.

In chronic stress conditions, excitatory transmission and intrinsic excitability are differentially regulated between D1- and D2-MSNs [50, 54, 57]. For example, D1-MSNs show increased intrinsic excitability in Sus mice after CSDS [51, 57, 58], which corresponds to the effects of *Shisa6* increase in Sus mice. This result might also be reflective of stress-induced reorganization of afferent circuits to D1-MSNs to selectively strengthen aversive afferent inputs, while at the same time decreasing connections from mPFC to NAc which promotes resiliency [59, 60]. A recent study shows that vHIP sends projections to the NAc and the circuit directly mediates SI behaviors [61]. More importantly, the vHIP-NAc glutamatergic circuit delivers aversive information only to D1-MSN, but not D2, and increased synaptic strength of the excitatory synapses directly increased depression-like behaviors [50]. These findings are well-aligned with D1-MSN specific increase of *Shisa6* and corresponding strengthening of AMPA currents at excitatory synapses including vHIP afferent synapses. Indeed, we show that downregulation of *Shisa6* in D1-MSNs reversed social avoidance behavior in socially defeated male mice, which validates causal relations between *Shisa6* and depressive phenotypes. On the other hand, overexpression of *Shisa6* prior or post to social defeat only exerted marginal effects on SI behaviors. We speculate that the loss of *Shisa6* function by knock-down might be more specific to the *Shisa6* expressing- and stress-relevant D1-MSN subsets rather than the gain of function in a broad population of D1-MSNs by *Shisa6* overexpression. Intriguingly, D1-MSN specific *Shisa6* overexpression without social stress exposure induced anhedonia in both male and female mice, while some opposing behavioral responses were observed in anxiety- or depression-like behavioral assays, which implicate possible sex specific roles of *Shisa6* in depression. Taken together, these data suggest that depressive phenotypes can be reversed by targeting *Shisa6*, as demonstrated in the *Shisa6* knock-down experiment.

Regulation of glutamate synapses is considered a potential target for antidepressants and our findings suggest that glutamatergic inputs to the NAc might be important in regulating depressive-like symptoms; given that we observe a selective increase of *Shisa6*, a bona fide AMPAR auxiliary subunit, in D1-MSNs. The regulation of *Shisa6*, an AMPAR regulatory protein, may act as a potential therapeutic target for treating depression. Indeed, postmortem tissue of depression patients show an increase in radio-ligand binding of AMPA in anterior cingulate cortex [62], and preclinical stress models show an increase in expression of AMPA receptor subunits following acute [63] and chronic [63–65] stress, while other studies find a decrease in expression of specific AMPA receptor subunits following stress [66–69]. Importantly, AMPA receptor antagonist NBQX infused into the NAc increased SI time, demonstrating that inhibition of AMPA receptor function induces an antidepressant-like response [51]. These data are consistent with our observation that *Shisa6* knock-down in D1-MSNs rescued social avoidance in socially defeated mice and show promising therapeutic applications of *Shisa6* in modulating depression.

In summary, we found *Shisa6* expression is increased in D1-MSNs of Sus mice and increases excitability of neurons, which modulates anxiety- and depression-like behaviors in mice. *Shisa6* is expressed in a cell-type and circuit-specific manner, and may directly alter the strength of pre-existing excitatory synapses related to aversive information flow. Taken together, *Shisa6* may be a critical link of dopamine and glutamatergic systems in the reward circuitry, and a promising molecular target for intervention during depression pathogenesis.

### Reporting summary

Further information on research design is available in the Nature Research Reporting Summary linked to this paper.

## Supplementary information

Supplementary legends

Supplementary figures 1–13

Supplementary tables 1–10

## Data Availability

RNA-seq data have been deposited to the NCBI GEO under accession number GSE147640.
