# Supplementary material for: Shisa6 mediates cell-type specific regulation of depression in the nucleus accumbens
Source: Mol Psychiatry. 2021 Jul 12;26(12):7316–27. doi: 10.1038/s41380-021-01217-8 (PMC8752624; doi:10.1038/s41380-021-01217-8)
Supplement: Supplementary file 2 — Supplementary figures 1–13 [file 41380_2021_1217_MOESM2_ESM.pdf]

Supplementary Figures 1-13

Supplementary Fig. 1

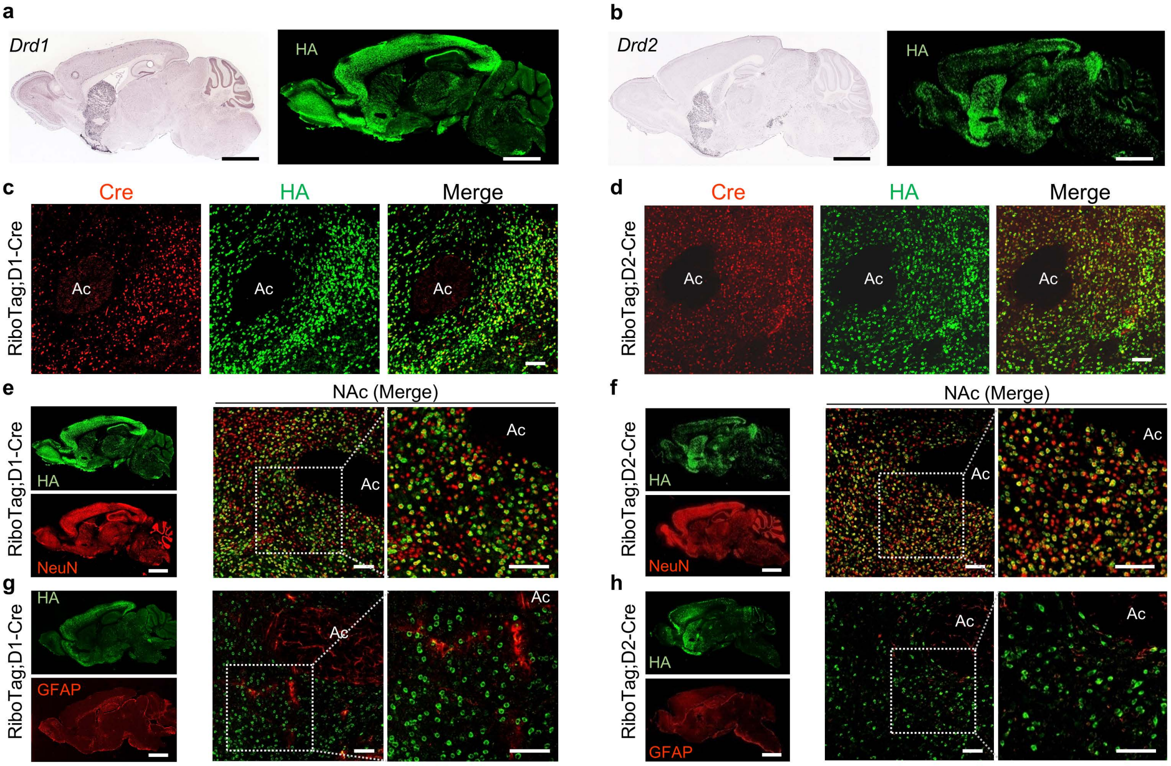

Supplementary Fig. 1 | Validation of RT; D1-Cre and RT; D2-Cre mice with immunohistochemistry. a, **b**, Brainwide comparisons between MSN subtype markers (Drd1 (**a**) or Drd2 (**b**); Allen mouse brain atlas, in situ hybridization data) and HA expression (immunohistochemistry) in sagittal sections (scale bar, 2 mm). **c**, **d**, Colocalization of Cre and HA expressions in RT; D1-Cre (**c**) and RT; D2-Cre mice (**d**) (coronal sections; scale bar, 100 µm). **e**-**h**, Neuron- or Glia-specific marker gene validation in RT; D1-Cre (**e**, **g**) and RT; D2-Cre mice (**f**, **h**) (Whole brain images: scale bar, 2 mm; merged images: scale bar: 100 µm).

Supplementary Fig. 2

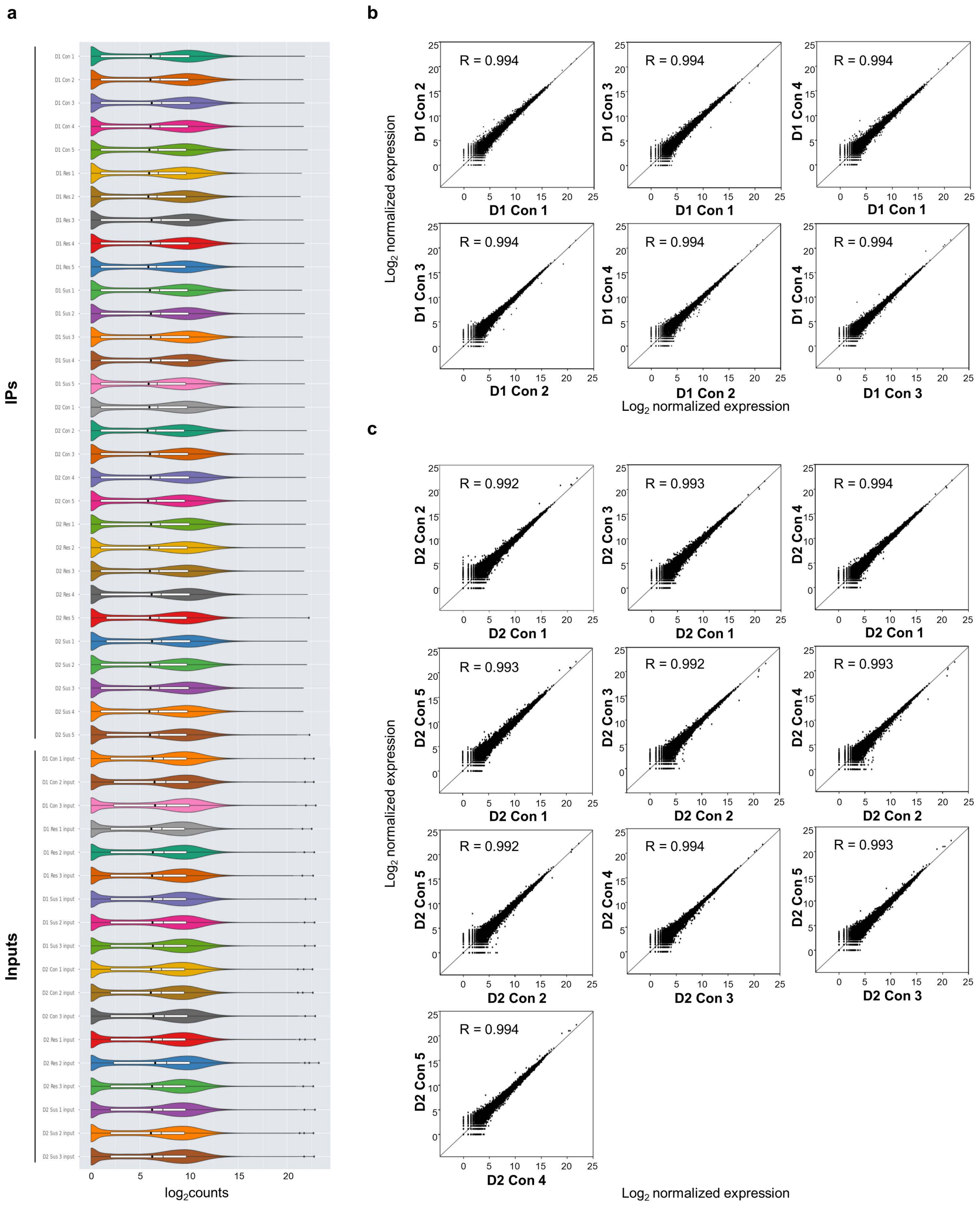

Supplementary Fig. 2 | RNA-seq quality control. a, Violin plots of un-normalized gene count distributions for RNA-seq samples. A box plot is overlaid on each violin plot to display minimum, first quartile, median, 3rd quartile and maximum. **b**, **c**, Intra-group variability between samples in D1- (**b**) or D2-MSN control group (**c**). Intra-group variability of each comparison was defined by Pearson's correlation coefficient.

Supplementary Fig. 3

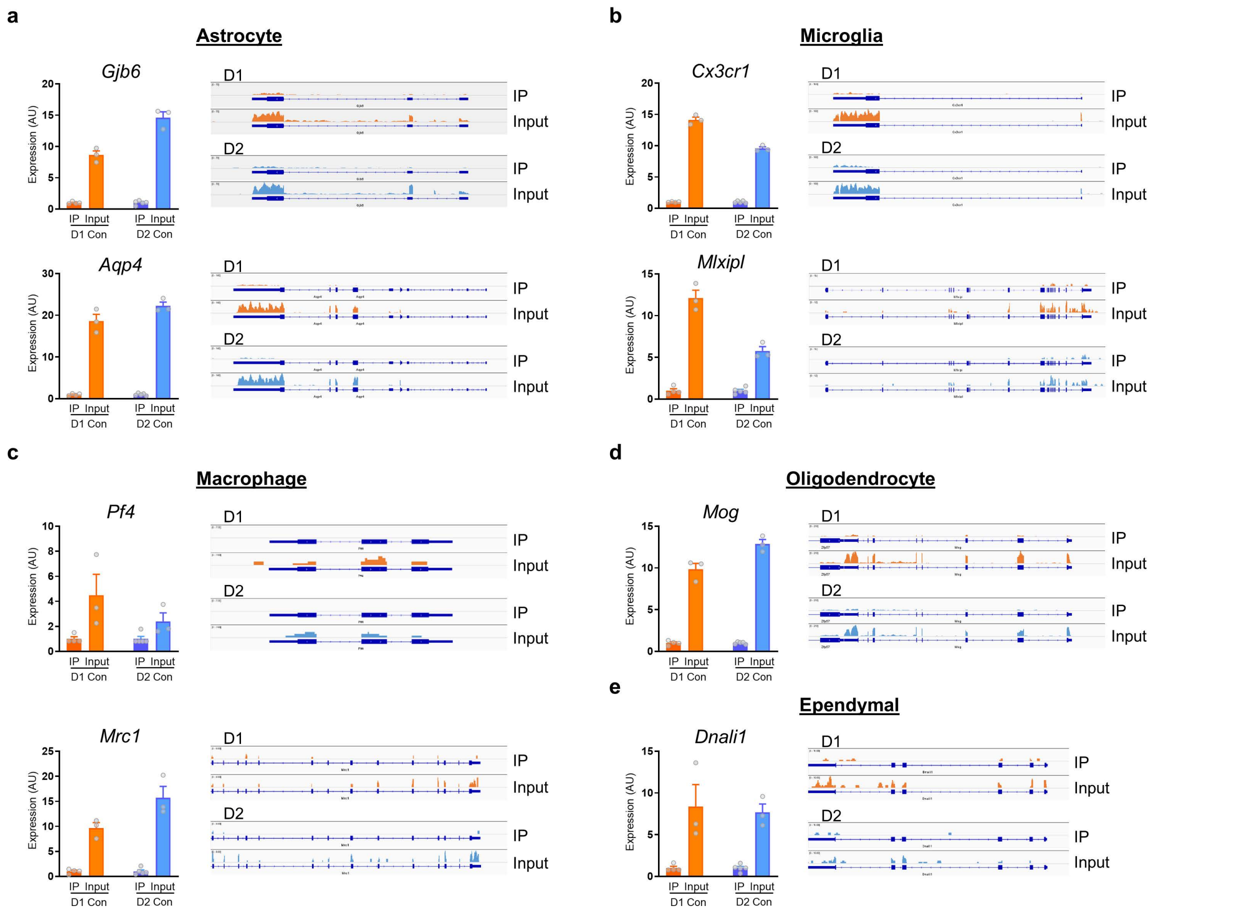

Supplementary Fig. 3 | Comparisons between RiboTag IP and input samples across non-neuronal cell types. Normalized expression levels and visualization of aligned reads in D1- and D2-MSNs for non-neuronal marker genes in astrocyte (**a**), microglia (**b**), macrophage (**c**), oligodendrocyte (**d**), and ependymal cells (**e**). Input samples show higher read alignment on non-neuronal marker genes (For IPs: D1, n=4; D2, n=5; For Inputs: D1, n=3; D2, n=3). Data are represented as the mean ± s.e.m.

Supplementary Fig. 4

a

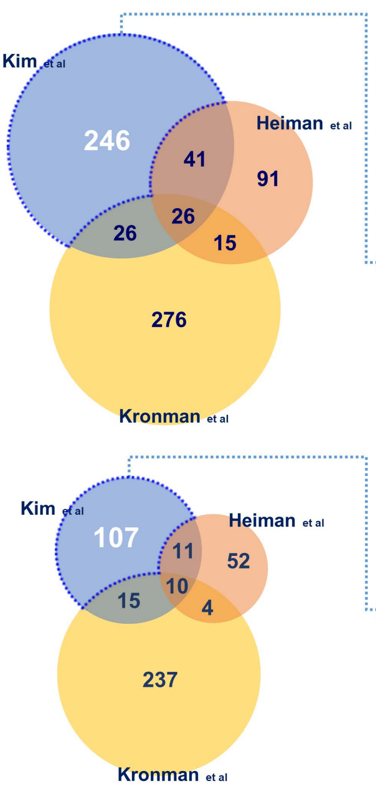

b

D1 enriched

D2 enriched

	Log_2 Fold diff.	Adjusted p-value	Average log_2 exp.
**Agt**	2.9597	4.58E-20	5.6535
**Oxtr**	1.6776	6.67E-15	7.9011
**Lingo2**	1.5357	1.9E-36	8.7347
**Ntng2**	1.5016	1.09E-13	8.4406
**il1rapl2**	1.3561	2.75E-06	7.1189
**Cntnap3**	1.1493	4.81E-19	9.1894
**Cntnap4**	0.9532	6.48E-10	8.9723
**Zfp703**	0.9108	3.47E-11	9.0163
**Atp6ap1l**	0.8880	5.68E-06	6.8973
**Fstl5**	0.8817	3.63E-09	9.6516
**Kcnj3**	0.8466	3.0E-08	10.4829
**Cnr1**	0.8317	2.06E-10	11.4653
**Kcnc2**	0.7603	5.2E-08	9.8291
**Grin3a**	0.7303	3.6E-07	10.2093
**Lingo1**	0.7226	2.55E-14	11.8912
**Adcy2**	0.6805	1.06E-10	10.7695
**Prkca**	0.6577	7.05E-08	12.4923
**Gabrg3**	-0.7950	0.000346	8.4209
**Th**	-0.8326	4.27E-11	8.0927
**Mfge8**	-0.8329	7.11E-13	10.1541
**Tshz1**	-0.9535	2.22E-23	11.5865
**Npy**	-1.0738	1.85E-11	8.6024
**Gpr52**	-1.1840	2.38E-26	10.6677
**Slc17a8**	-1.2562	2.54E-12	8.3058
**Oprd1**	-1.3151	5.21E-24	10.0032
**Calcr**	-1.4718	5.53E-20	8.6474
**Tacr1**	-1.8479	1.09E-74	9.1423
**Mdfic**	-1.9550	4.94E-24	7.4359
**Chat**	-3.6008	1.76E-104	8.3439
**Ntrk1**	-3.8610	6.36E-70	7.0785

c

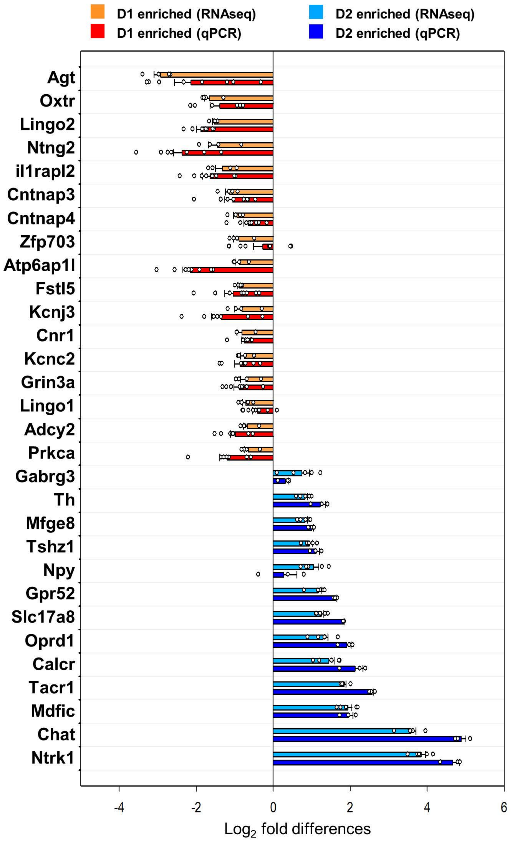

d

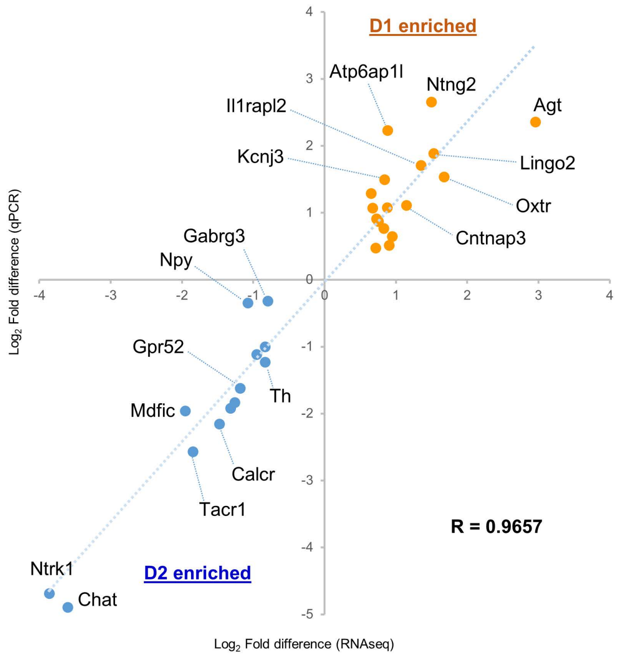

e

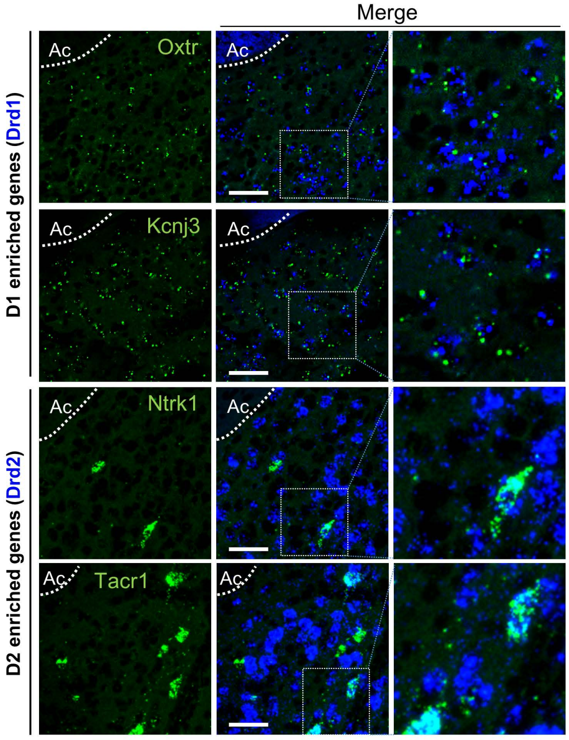

Supplementary Fig. 4 | Meta-analysis of D1- and D2-specific genes detected from MSN population based studies and novel MSN subtype specific genes. a, Venn diagram comparisons of D1- or D2-enriched gene lists from current data (Kim et al.; [Fold change (FC)]>1.5, adjusted *p*-value < 0.05), Heiman et al. ([Fold change (FC)]>1.5, adjusted *p*-value < 0.1) and Kronman *et al*. (adjusted *p*-value < 0.05). Numbers indicate the number of genes included in each region of venn diagrams. **b-d**, Novel MSN subtype specific DEGs detected from RNA-seq (D1 enriched, n=4; D2 enriched, n=5) (**b**) and additional qPCR validation with independent samples (D1 enriched, n=8; D2 enriched, n=3) in bar chart (**c**) and scatter plot (**d**; Pearson's correlation, R=0.9657). **e**, Validation of selected DEG expression (*Oxtr*, *Kcnj3*, *Ntrk1*, and *Tacr1*) by *in situ* hybridization (RNAscope; scale bar, 50 µm).

Supplementary Fig. 5

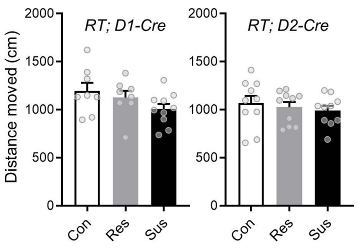

Supplementary Fig. 5 | Locomotor activity of socially defeated mice used for transcriptome analysis. Locomotor activity data from no target session (2.5 min) of each social interaction test, which correspond to Fig. 3c.

Supplementary Fig. 6

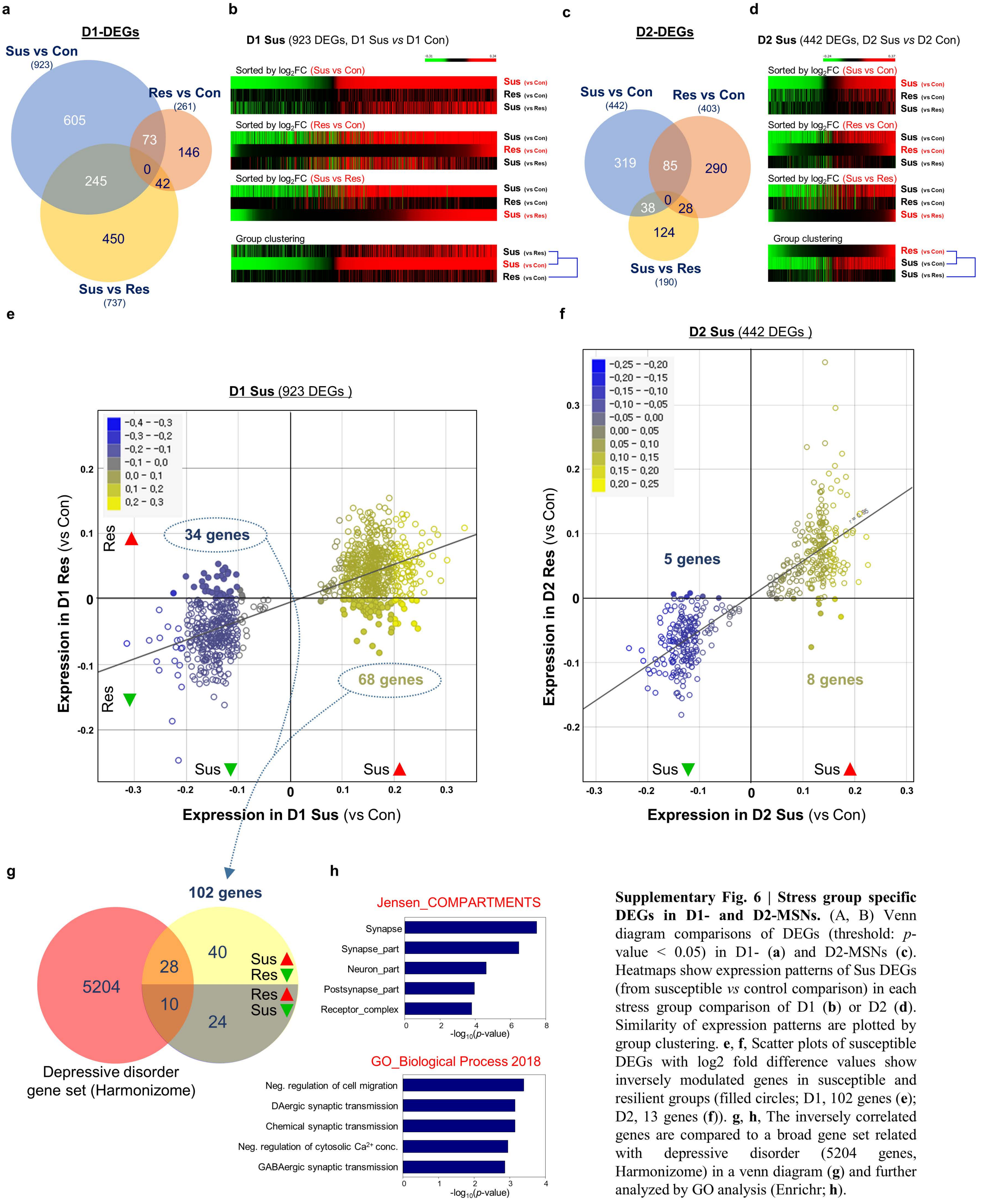

Supplementary Fig. 6 | Stress group specific DEGs in D1- and D2-MSNs. (A, B) Venn diagram comparisons of DEGs (threshold: *p*-value < 0.05) in D1- (**a**) and D2-MSNs (**c**). Heatmaps show expression patterns of Sus DEGs (from susceptible *vs* control comparison) in each stress group comparison of D1 (**b**) or D2 (**d**). Similarity of expression patterns are plotted by group clustering. **e**, **f**, Scatter plots of susceptible DEGs with log2 fold difference values show inversely modulated genes in susceptible and resilient groups (filled circles; D1, 102 genes (**e**); D2, 13 genes (**f**)). **g**, **h**, The inversely correlated genes are compared to a broad gene set related with depressive disorder (5204 genes, Harmonizome) in a venn diagram (**g**) and further analyzed by GO analysis (Enrichr; **h**).

Supplementary Fig. 7

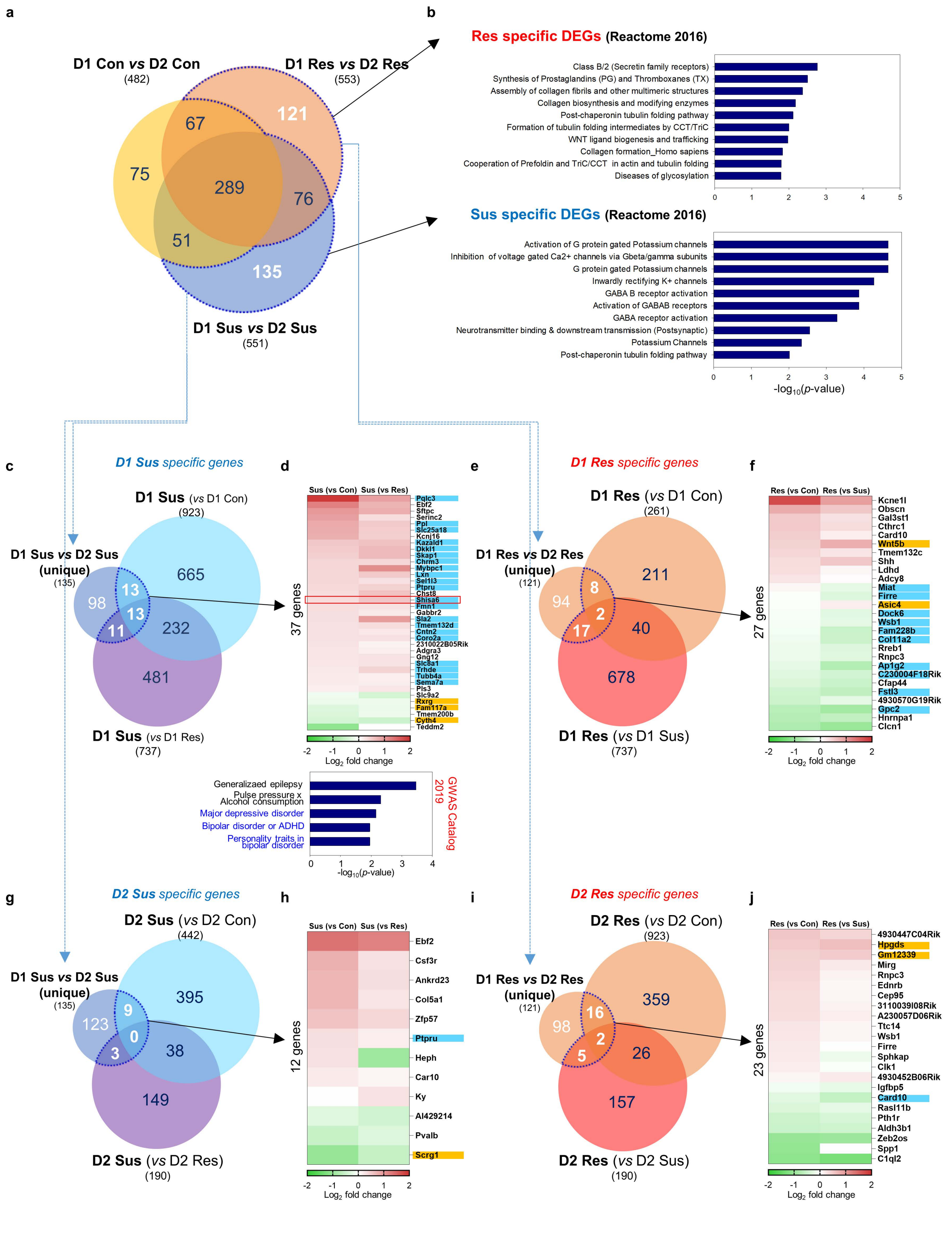

Supplementary Fig. 7 | MSN-subtype specific DEGs in each stress group and identification of resilient and susceptible specific genes. a, **b**, Comparisons between D1 and D2 gene expression data for each stress group (D1 vs D2; threshold: [Fold change (FC)]>1.5, adjusted *p*-value < 0.05). Venn diagram of stress group specific DEGs (**a**). GO analyses of resilient (Res; upper panel)- and susceptible (Sus; lower panel)-unique DEGs (**b**). Numbers indicate the number of genes included in each region of venn diagrams. **c**, **d**, D1-Susceptible specific gene list (37 genes) from a venn diagram comparison. The susceptible unique 135 DEGs (D1 vs D2) are compared with susceptible DEG lists from D1-MSN data (either *vs* control or *vs* resilient group comparisons in D1; Fig. 3d) to detect D1-specific DEGs (**c**). From the comparison, 37 genes are identified as D1-susceptible specific DEGs and *Shisa6* is indicated in a red rectangle (**d**). GO analysis of the 37 genes detected three depression relevant terms in top 5 (in blue letters; lower panel in **d**) **e**, **f**, D1-resilient specific gene list (27 genes). **g**, **h**, D2-susceptible specific gene list (12 genes). **i**, **j**, D2-resilient specific gene list (23 genes). Numbers indicate the number of genes included in each region of venn diagrams. Genes with significant correlation to social interaction (SI ratio) are highlighted with cyan (inverse correlation, $p < 0.05$) or orange (positive correlation, $p < 0.05$) (# of genes with significant correlation; D1 Sus: 24 of 37 genes, 64.9% (**d**); D1 Res: 12 of 27 genes, 44.4% (**f**); D2 Sus: 2 of 12 genes, 16.7% (**h**); D2 Res: 3 of 23 genes, 13.0%(**j**)).

Supplementary Fig. 8

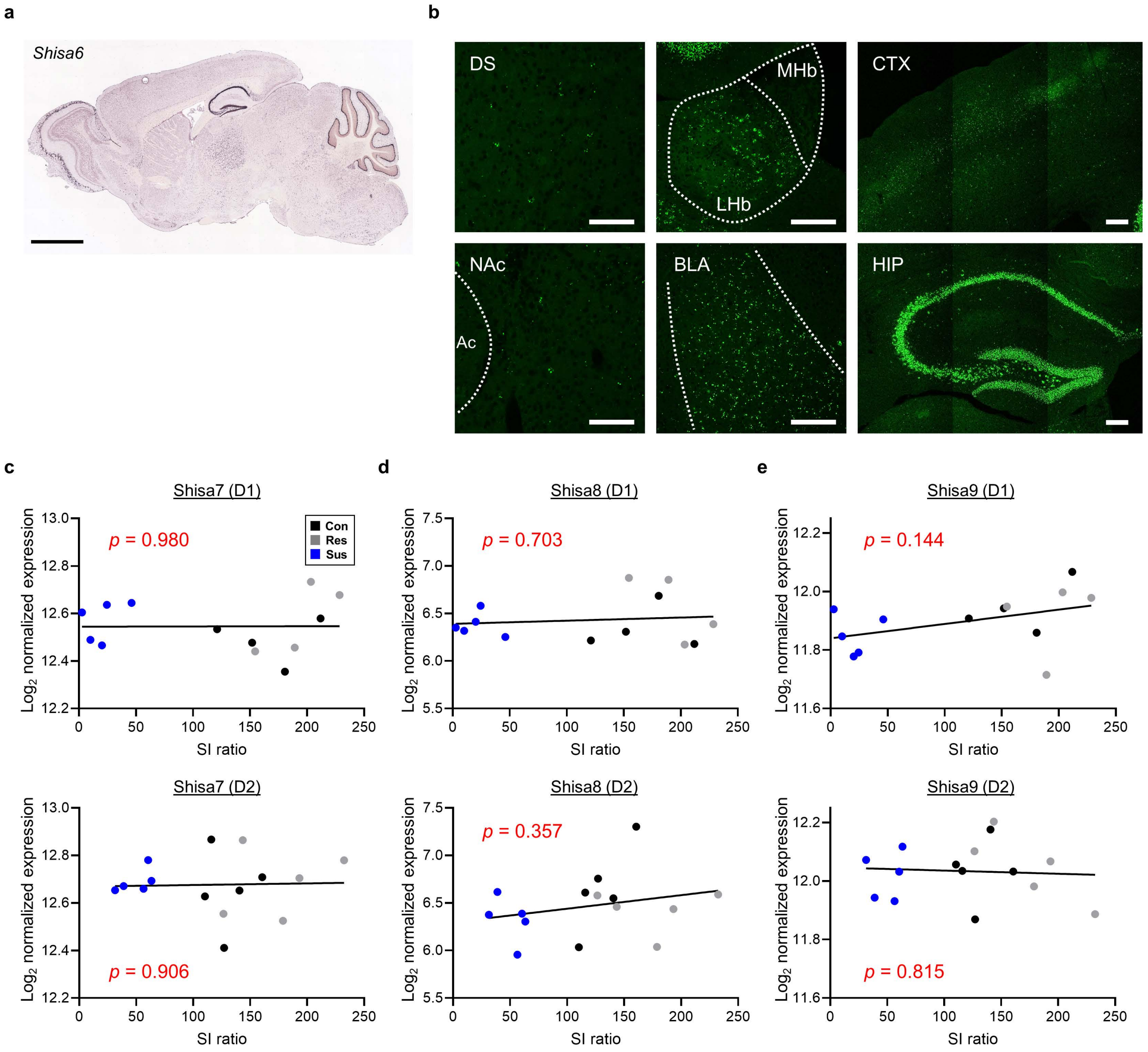

Supplementary Fig. 8 | Validation of *Shisa6* expressions in various brain regions and behavioral correlation of Shisa (CKAMP subfamily) expressions in D1- and D2-MSNs. a, *Shisa6* expression data in Allen mouse brain atlas (sagittal section; scale bar, 2 mm). **b**, *Shisa6* expression data in various brain regions by RNAscope. DS, dorsal striatum; LHb, lateral habenula; MHb, medial habenula; CTX, cerebral cortex; NAc, nucleus accumbens; AC, anterior commissure; BLA, basolateral amygdala; HIP, hippocampus (scale bar, 200 µm). **c**-**e**, Social interaction data were plotted with gene expression levels of corresponding D1 or D2 samples: *Shisa7* (c), *Shisa8* (d), and *Shisa9* (e). For experiments in **c**-**e**, D1 Con, n=4; D1 Res, n=4; D1 Sus, n=5; D2 Con, n=5; D2 Res, n=5; D2 Sus, n=5. *p*-values in linear regression.

Supplementary Fig. 9

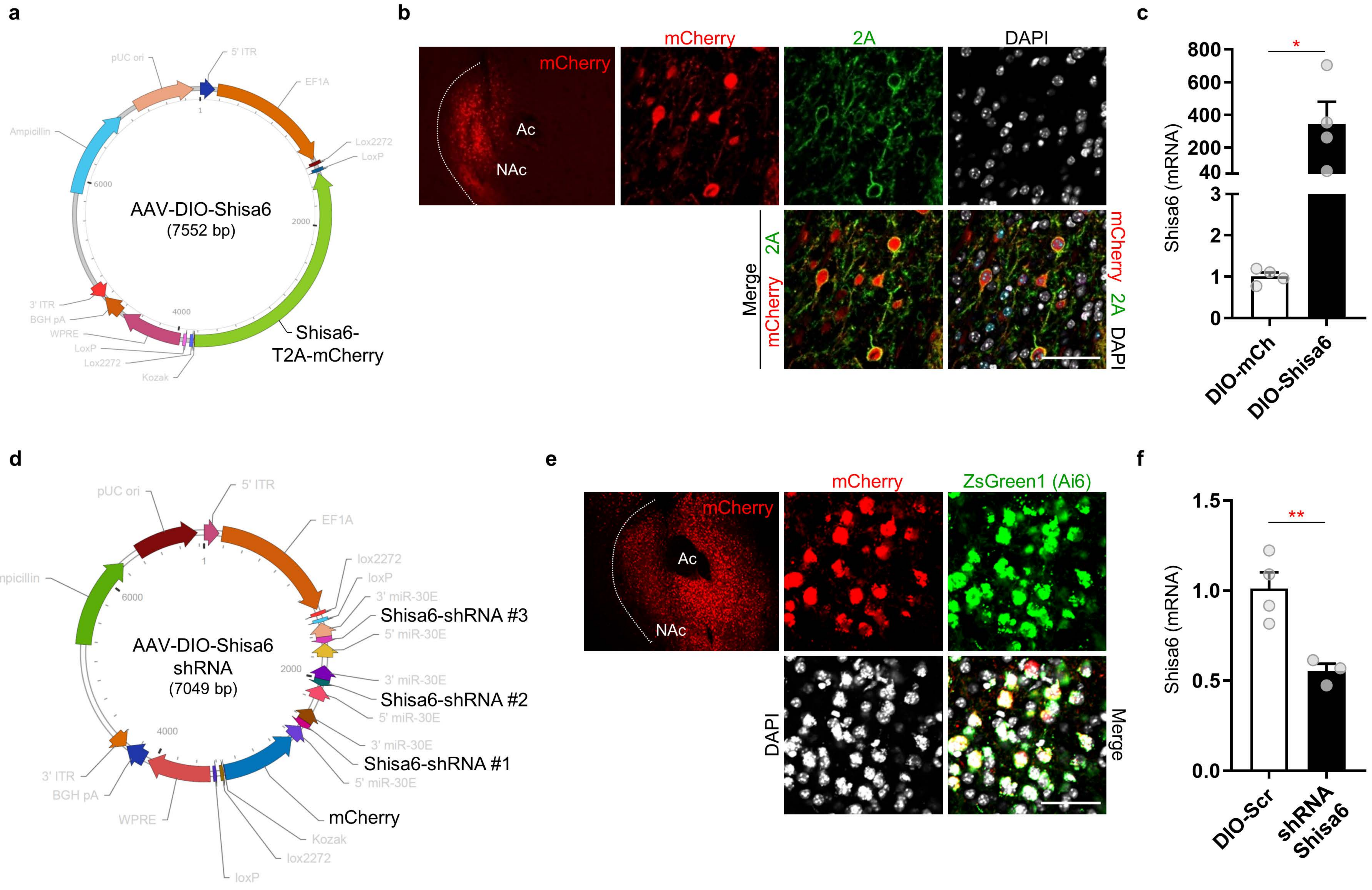

Supplementary Fig. 9 | Cre-dependent Shisa6 expressing- and Shisa6 shRNA-AAV constructs. a Schematics of AAV-DIO-Shisa6 construct. Shisa6 coding sequence is linked to mCherry cassette via self-cleaving thosea asigna virus 2A (T2A) sequence. **b**, Validation of D1-MSN specific viral expression of Shisa6 by immunohistochemistry. The self-cleavage of T2A peptide leaves 20 amino acids from T2A at the C-terminus of SHISA6, and the 2A peptide tagged SHISA6 can be detected with an anti-2A peptide antibody. Scale bar, 50 µm. **c**, qPCR validation of Shisa6 overexpression by AAV-DIO-Shisa6 (n=4). **d**, Schematics of AAV-DIO-Shisa6 shRNA construct. Three Shisa6 shRNA sequences were inserted in miR-30E shRNA cassette. **e**, Validation of D1-MSN specific expression of AAV-DIO-Shisa6 shRNA by immunohistochemistry. Scale bar, 50 µm. **f**, qPCR validation of Shisa6 knock-down by AAV-DIO-Shisa6 shRNA and scrambled (n=3-4). Graphs are represented as mean ± s.e.m. (**p*<0.05; ***p*<0.01; t-test, one-tailed (**c, f**)).

Supplementary Fig. 10

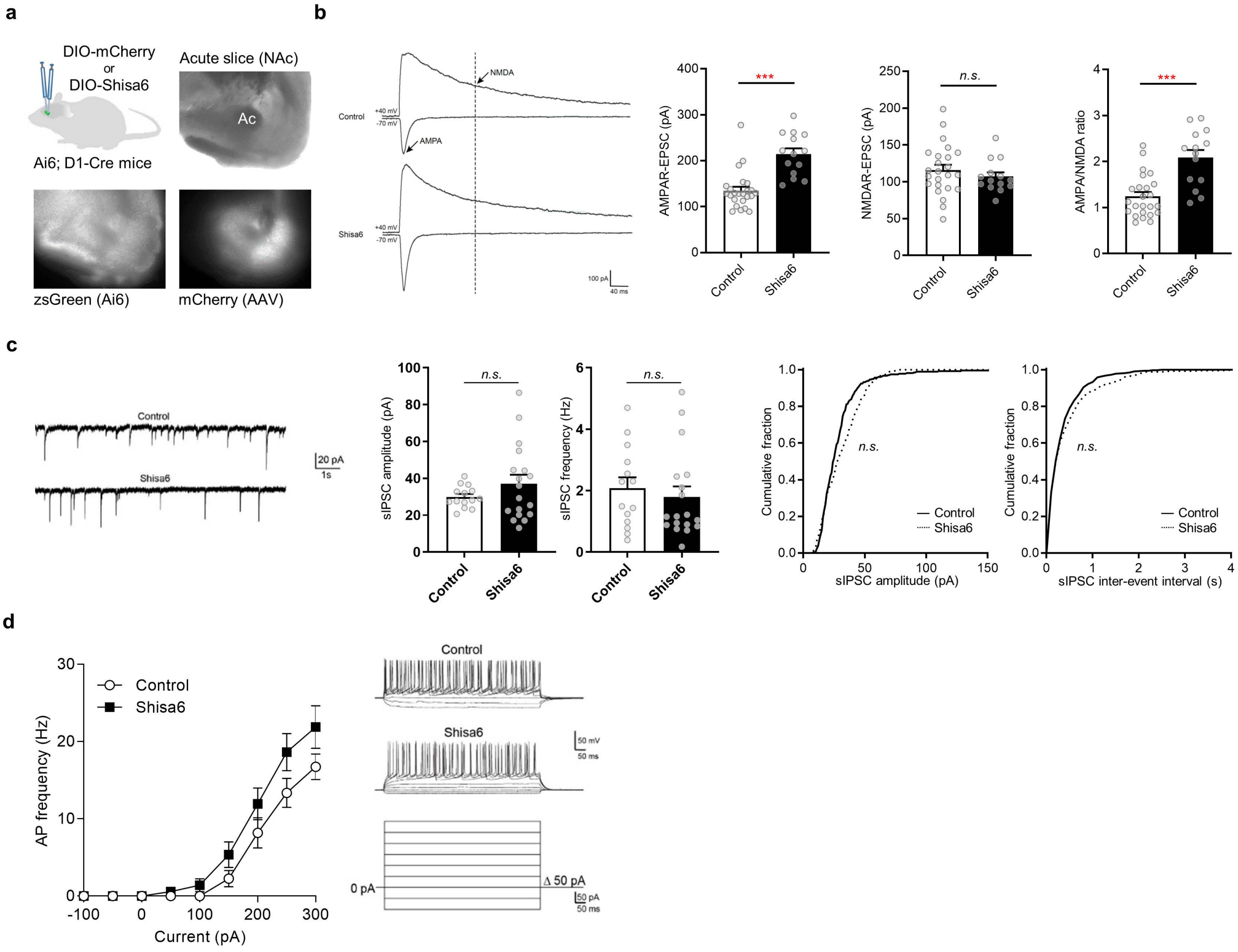

Supplementary Fig. 10 | Electrophysiological analysis of Shisa6 expressing D1-MSNs. a, Schematics of acute slice preparation. **b**, Representative AMPAR- and NMDAR-EPSC traces, bar graphs (mean ± s.e.m.; AMPAR-EPSC: control, 134.7 ± 8.3 pA, Shisa6: 214.5 ± 12.4 pA; NMDAR-EPSC: control, 116.1 ± 7.0 pA; Shisa6, 107.2 ± 5.8 pA; AMPA/NMDA ratio: control, 1.244 ± 0.093, Shisa6: 2.086 ± 0.165; control, n=24; Shisa6, n=14). **c**, Representative sIPSC traces, bar graphs (mean ± s.e.m.) and cumulative distribution of sIPSC amplitude and frequency in D1-MSN from control versus Shisa6 overexpressed mice (amplitude: control, 30.0 ± 1.6 pA, Shisa6: 37.1 ± 4.8 pA; frequency: control, 2.1 ± 0.4 Hz; Shisa6, 1.8 ± 0.3 Hz; control, n=14; Shisa6, n=18). **d**, Shisa6 overexpression does not affect action potential (AP) frequency of D1-MSNs. Left: Action potential frequency (Hz) curve of control and Shisa6 overexpressed neurons following incremental current injection. Right: Sample current-clamp recordings of action potentials from D1-MSNs neurons following injection of depolarizing currents (pA). Data are represented as the mean ± s.e.m. (***p<0.001; n.s., not significant; t-test, two-tailed (**b**, **c**); two-way ANOVA with Sidak's post-hoc test (**d**))

Supplementary Fig. 11

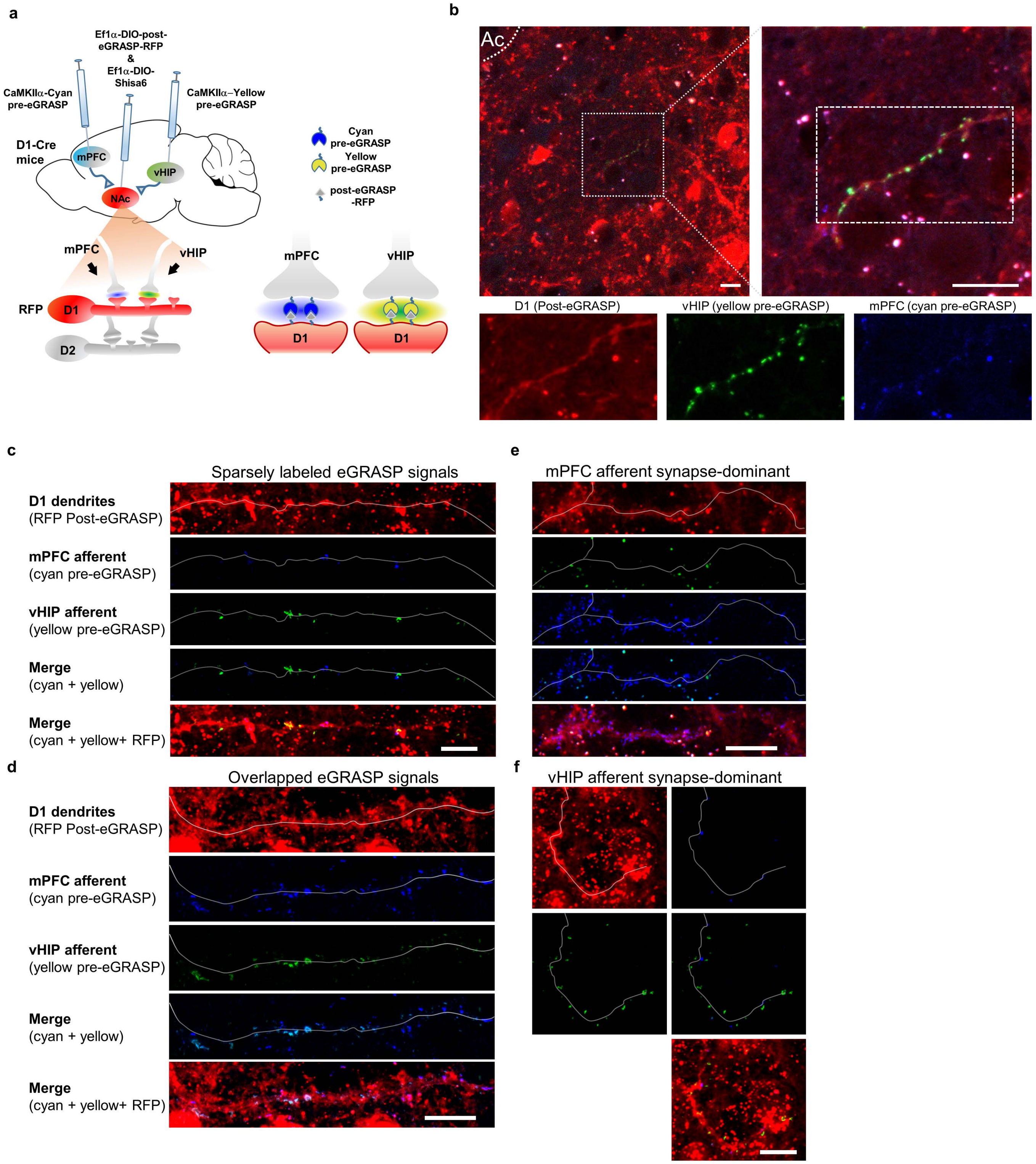

Supplementary Fig. 11 | Afferent input specific synapse labeling of D1-MSN using dual-eGRASP. a, Schematic diagrams of D1-MSN specific eGRASP labeling in the NAc. **b**, Identification of eGRASP in the NAc coronal sections. **c-f**, Additional eGRASP images of D1-dendrite in the NAc showing various afferent innervation density and pattern. Dendrites with eGRASP signals are traced in gray lines. Scale bar, 10 μm.

Supplementary Fig. 12

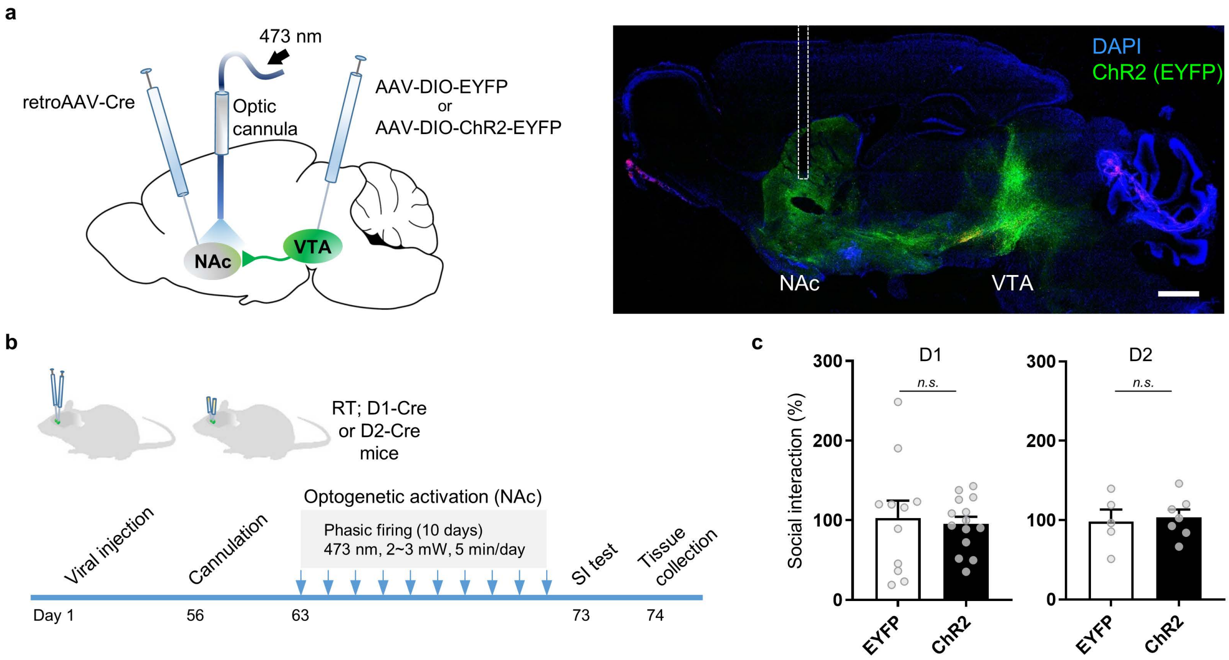

Supplementary Fig. 12 | Pro-susceptible optogenetic stimulation in the VTA to NAc circuitry. a, AAV injection scheme and representative image of ChR2-EYFP labeled VTA to NAc circuit (cannula tract outlined by dashed line; scale bar, 1 mm). Only VTA neurons which project to the NAc were labeled with ChR2-EYFP or EYFP. **b**, Schematics of optogenetic experiments. **c**, Social interaction (SI) test after chronic activation of the VTA to NAc circuit. The 10 day-optogenetic activation did not change social interaction in ChR2 expressing mice (D1: EYFP, n=11; ChR2, n=14; D2: EYFP, n=5; ChR2, n=7). Data are represented as mean ± s.e.m. (n.s., not significant; t-test, two-tailed).

Supplementary Fig. 13

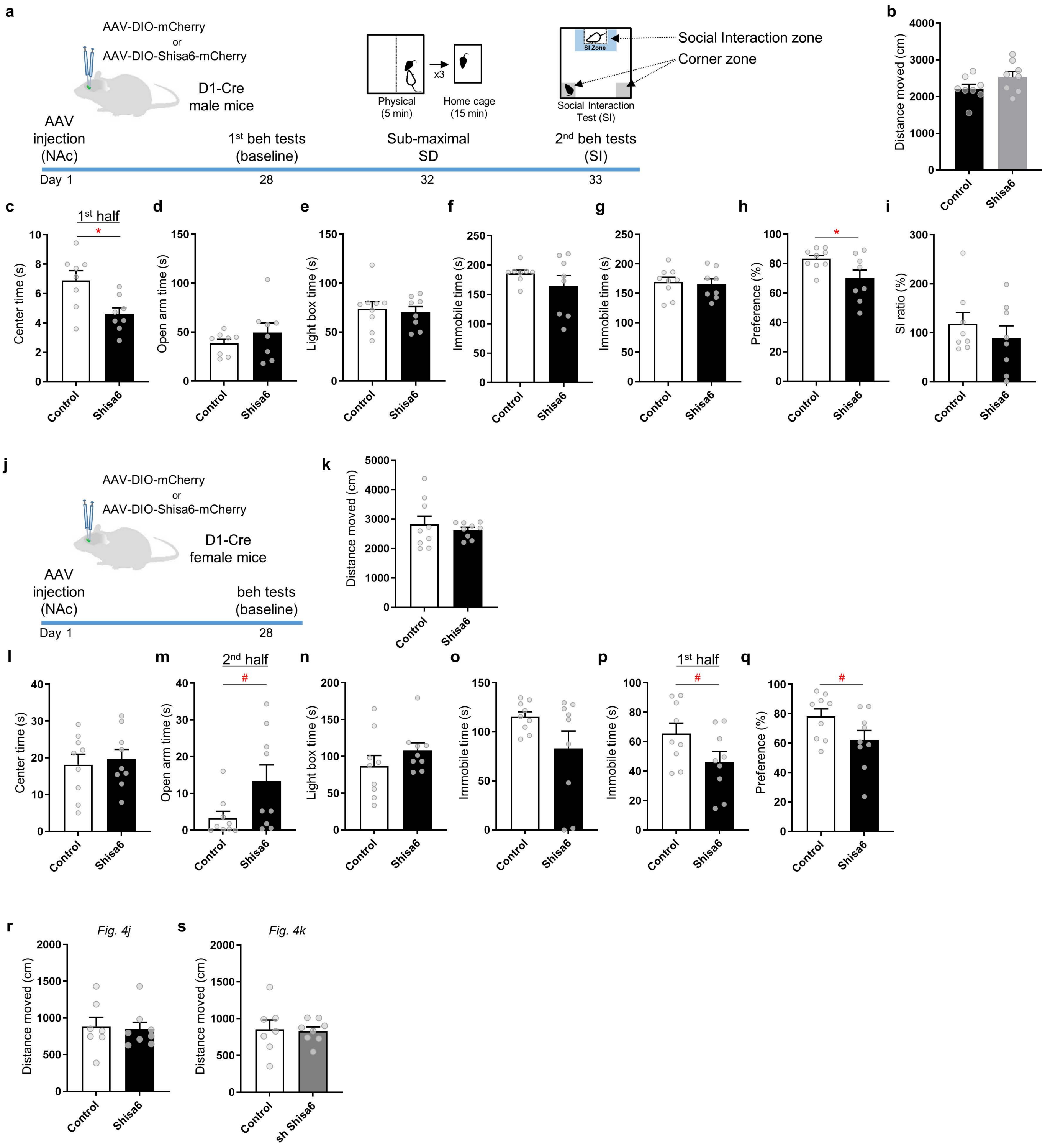

Supplementary Fig. 13 | Anxiety- and depression-like behaviors in D1-MSN specific Shisa6 overexpressed male and female mice. a, Experimental timeline and schematic of baseline behavioral testing and sub-maximal stress paradigm of male mice (**b-i**; Control, n=8-9; Shisa6, n=8). **b-h**, Baseline behavior tests for anxiety- (**b**, locomotor (open field); **c**, open field; **d**, elevated plus maze; **e**, light-dark box) and depression-like behaviors (**f**, forced swim test; **g**, tail suspension test). Testing anhedonia with sucrose preference test (**h**). **i**, Social interactions (SI) after submaximal social defeat. **j**, Experimental timeline and schematic of baseline behaviors of female mice (**k-q**; Control, n=9; Shisa6, n=9). **k-p**, Baseline behavior tests for anxiety- (**k**, locomotor (open field); **l**, open field; **m**, elevated plus maze; **n**, light-dark box) and depression-like behaviors (**o**, forced swim test; **p**, tail suspension test). Testing anhedonia with sucrose preference test (**q**). **r**, **s**, Locomotor activity data from no target session (2.5 min) of each social interaction test, which correspond to Fig. 4j (**r**) and Fig. 4k (**s**). Data were analyzed in time bins of 2.5 min (1st and 2nd half; labeled) and full time (5 min) and significant results were presented (otherwise, full time results were presented). Graphs are represented as mean ± s.e.m. ($^*p<0.05$; $^{\#}p<0.08$; t-test, two-tailed).
